# Defect Driven Electronic Structure Reconfiguration and Hierarchical Phonon Suppression in Ag_2_Se Thermoelectrics

**DOI:** 10.1002/advs.75408

**Published:** 2026-04-21

**Authors:** Yineng Gou, Mengyao Li, Fang Lyu, Xiaolong Sun, Muyang Huang, Wenhao Xie, Liangwei Hu, Wei Cao, Yue Hou, Yong Liu, Andreu Cabot, Ziyu Wang

**Affiliations:** ^1^ School of Physics and Technology Wuhan University Wuhan P. R. China; ^2^ School of Integrated Circuits Wuhan University Wuhan P. R. China; ^3^ School of Physics and Laboratory of Zhongyuan Light Zhengzhou University Zhengzhou P. R. China; ^4^ Key Laboratory of Artificial Micro‐and Nano‐structures of Ministry of Education and School of Physics and Technology Wuhan University Wuhan P. R. China; ^5^ Catalonia Institute for Energy Research‐IREC Sant Adrià de Besòs Barcelona Spain; ^6^ ICREA Barcelona Catalonia Spain

**Keywords:** Ag_2_Se, decoupling, substitution, thermoelectric

## Abstract

Ag_2_Se is a promising n‐type thermoelectric for near‐room‐temperature applications. Nevertheless, further enhancement of its figure of merit (*ZT*) demands a delicate balance between electronic and phononic transport. Here, we develop a facile and controllable wet‐chemical strategy under ambient conditions to synthesize orthorhombic Ag_2_Se nanocrystals. Indium substitution at the Ag site modulates the electronic structure, leading to a Fermi‐level upshift, band‐gap narrowing, and increased carrier concentration, thereby enhancing electrical transport. Meanwhile, In's incorporation introduces local dislocations and anisotropic strain fields that induce lattice softening and reduce the sound velocity, resulting in enhanced phonon scattering and suppressed lattice thermal conductivity. First‐principles calculations further reveal that In incorporation weakens Ag─Se antibonding interactions and suppresses Ag s‐d hybridization in favor of In s‐p hybridization, thereby mitigating potential fluctuations and enhancing carrier mobility. Benefiting from this synergistic phonon‐electron optimization, Ag_1.9287_In_0.0713_Se achieves a high power factor of ∼3100 µW m^−^^1^ K^−^^2^ and a peak *ZT* of ∼1.2 at 398 K. Prototype modules exhibit consistently higher output voltage and power density, while hardness mapping confirms improved lattice resilience. This work highlights the potential of coupling defect chemistry with electronic structure engineering as an effective strategy to advance high‐performance and mechanically robust Ag_2_Se ‐based thermoelectrics.

## Graphical abstract

Indium substitution in Ag_2_Se nanocrystals simultaneously tailors electronic structure and lattice dynamics. Fermi‐level upshift and band‐gap narrowing enhance electrical transport, while defect‐induced lattice softening intensifies phonon scattering and suppresses lattice thermal conductivity. This synergistic phonon‐electron optimization enables high thermoelectric performance, delivering a peak ZT of ∼1.2 at 398 K with improved mechanical robustness.

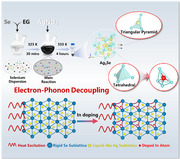

## 1 Introduction

Thermoelectric (TE) materials enable the direct conversion of heat into electricity, offering promising applications in solid‐state cooling and waste heat recovery [1, 2, 3, 4, 5, 6]. The energy conversion efficiency of a TE material is evaluated by the dimensionless figure of merit, *ZT* = *S^2^σT*/*κ_tot_*, where *S* is the Seebeck coefficient, *σ* is the electrical conductivity, *κ_tot_* is the total thermal conductivity, and *T* is the absolute temperature. Achieving high *ZT* values therefore, requires the simultaneous optimization of electrical and thermal transport properties.

PLEC, referred to as Phonon‐Liquid Electron‐Crystal, is a special transport regime in certain thermoelectrics where phonons behave in a liquid‐like manner, while electrons propagate through a well‐ordered crystalline framework. In PLEC materials, one sublattice (typically composed of mobile cations such as Cu^+^ or Ag^+^) exhibits liquid‐like diffusion or strong anharmonic vibrations, which severely scatter heat‐carrying phonons and thus lead to ultralow lattice thermal conductivity [7, 8, 9]. Meanwhile, the remaining rigid framework maintains long‐range crystallinity, enabling high electrical conductivity and favorable carrier transport, similar to that of conventional crystalline solids. Conventional PLEC materials are predominantly superionic chalcogenides in which a subset of cations (most commonly Cu^+^ or Ag^+^) exhibits highly dynamic, liquid‐like motion at elevated temperatures, while the anion framework remains structurally ordered. This intrinsic sublattice decoupling leads to strong phonon scattering and ultralow lattice thermal conductivity, as heat‐carrying phonons are disrupted by the anharmonic and diffusive cation dynamics. At the same time, the preserved long‐range order of the rigid framework enables band‐like electronic transport, maintaining favorable electrical conductivity and carrier mobility. Representative and widely accepted conventional PLEC systems include Cu_2_X (X = S, Se, Te) [10, 11, 12, 13], Ag_2_X (X = S, Se, Te) [14, 15, 16] and related layered compounds such as CuCrSe_2_ [17] and AgCrSe_2_ [18], which are frequently used as benchmark platforms for understanding and extending the PLEC concept in thermoelectric materials design. Among the various TE material systems, silver selenide (Ag_2_Se) has attracted considerable attention due to its distinctive “phonon liquid‐electron crystal” (PLEC) characteristics [19, 20, 21]. Ag ions exhibit dynamic liquid‐like diffusion, which introduces strong anharmonicity and selective phonon scattering that suppresses the lattice thermal conductivity toward the minimum. With liquid‐like sublattices, transverse acoustic phonons can collapse while longitudinal modes survive, leading to intrinsically ultralow κ_1_ in a crystalline solid. In contrast, electronic transport is predominantly governed by the rigid Se sublattice that forms the conduction band edge and provides relatively stable pathways for charge carriers [22, 23]. This unique phonon‐electron decoupling mechanism makes Ag_2_Se one of the most promising n‐type thermoelectric candidates near room temperature [24].

Nevertheless, the intrinsic electrical performance of Ag_2_Se, particularly its power factor (*PF* = *S^2^σ*), remains insufficient, limiting further enhancement of *ZT* [25]. To address this challenge, a range of material modification strategies has been explored, including elemental doping, band engineering, and composite construction [26, 27, 28, 29, 30, 31, 32, 33, 34, 35, 36, 37, 38]. Among them, composite construction introduces interface scattering that effectively reduces thermal conductivity but often at the expense of electrical transport. Band engineering can enhance the power factor by tailoring the band structure, yet it is technically complex and difficult to precisely control. In particular, conventional elemental doping predominantly regulates carrier concentration and therefore typically follows the inherent *σ‐S* trade‐off described by the Pisarenko relation. Consequently, simple carrier doping alone rarely breaks this coupling. To address this fundamental constraint, various decoupling strategies have been developed, including band structure engineering (e.g., band convergence and resonant levels), defect structure engineering, and hierarchical phonon scattering architectures. These approaches aim to manipulate the electronic density of states and phonon transport pathways beyond the limits of rigid‐band behavior. Within this context, substitutional doping, when judiciously designed, can serve not merely as a carrier regulator but as a means of concurrently modulating electronic structure and lattice dynamics, thereby enabling synergistic optimization of thermoelectric performance [39, 40, 41].

In this work, we employ a controllable wet‐chemical synthesis that may provide better structural and compositional control to obtain In‐doped Ag_2_Se powders, followed by spark plasma sintering to fabricate dense bulk samples. We demonstrate that substitution provides a dual optimization pathway for Ag_2_Se thermoelectrics. On the phononic side, local dislocations, anisotropic strain fields, and lattice softening scatter phonons collectively over multiple length scales, leading to a pronounced reduction in lattice thermal conductivity. On the electronic side, first‐principles calculations reveal that In incorporation weakens Ag─Se antibonding, suppresses Ag s‐d hybridization in favor of In s‐p coupling, and narrows the band gap, thereby reducing potential fluctuations and enhancing carrier mobility. These synergistic effects enable Ag_1.9287_In_0.0713_Se to achieve a peak *ZT* of ∼1.2 at 398 K, together with robust mechanical properties.

## 2 Results and Discussion

**FIGURE 1:**
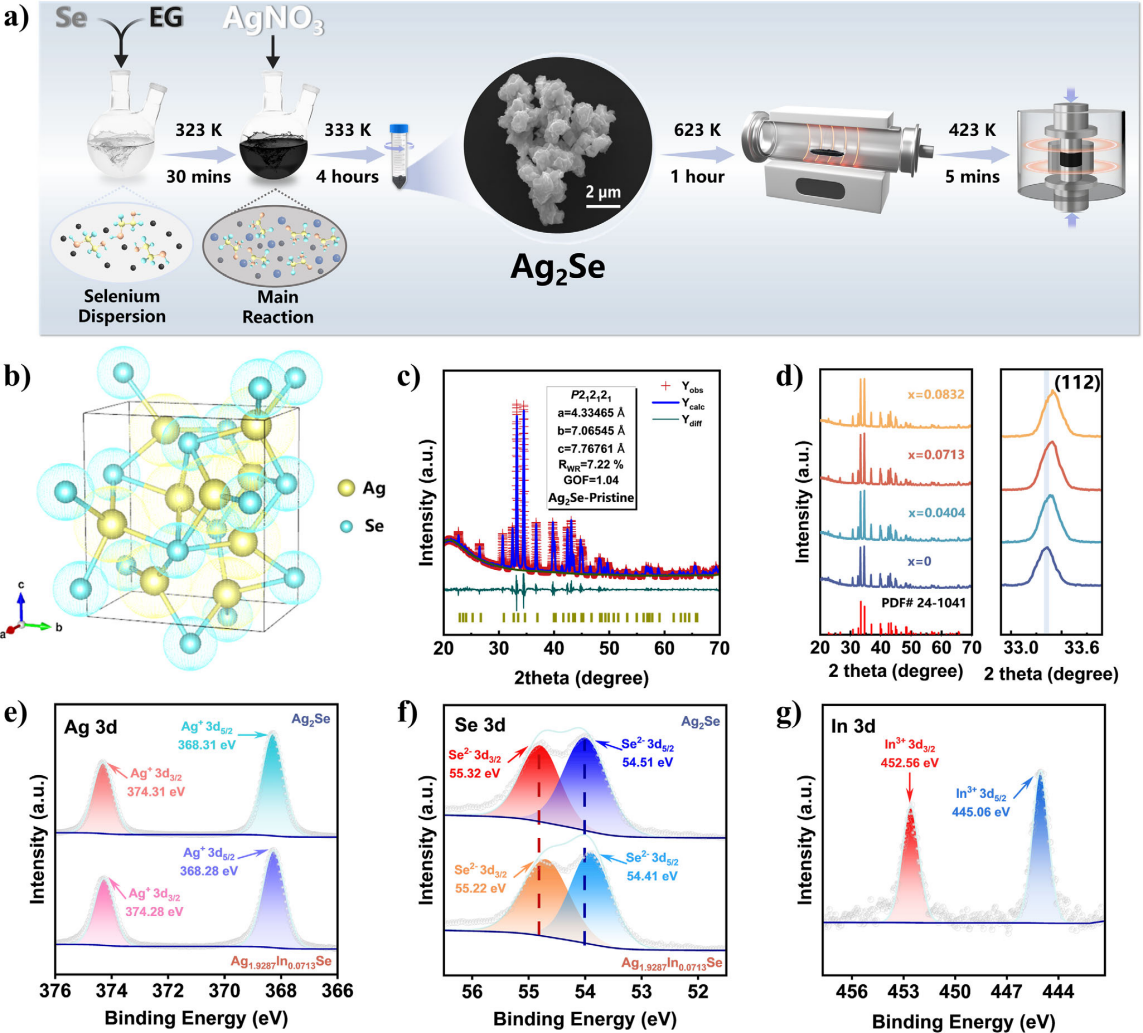
(a) Schematic illustration of the fabrication of Ag_2‐x_In_x_Se powder samples. (b) Crystal Structure of pristine Ag_2_Se with Ag atom in yellow and Se atom in blue. (c) Rietveld refinement of the XRD pattern of Ag_2_Se. (d) XRD patterns of Ag_2‐x_In_x_Se. (e–g) XPS spectra of Ag 3d, In 3d, and Se 3d of Ag_1.9287_In_0.0713_Se sample.

Silver selenide nanocrystals were synthesized through a simple wet‐chemical method, as detailed in the Supporting Information, and the fabrication process is schematically illustrated in Figure 1a. The orthorhombic Ag_2_Se crystal structure contains two distinct Ag coordination environments: one at the body‐centered position of a tetrahedral void and the other at the apex of a triangular pyramid (Figure 1b). To optimize the thermoelectric performance, Ag_2‐x_In_x_Se (x = 0, 0.0404, 0.0713, 0.0832) samples were prepared, with the In concentrations confirmed by inductively coupled plasma‐optical emission spectroscopy (ICP‐OES) analysis (Table S1).

X‐ray diffraction (XRD) patterns of as‐synthesized Ag_2‐x_In_x_Se powder (Figure 1d) confirm a phase‐pure orthorhombic Ag_2_Se phase (JCPDS No. 24–1041) without detectable secondary phases. With increasing In content, the (112) diffraction peak gradually shifts to higher angles, indicative of lattice contraction. Rietveld refinements (Figure 1c; Figure S1) further demonstrate that all Ag_2‐x_In_x_Se powder samples retain the orthorhombic *P2_1_2_1_2_1_* structure with satisfactory reliability factors (R_WR_ ≈ 6.5‐7.8%, GOF ≈ 1.0). The refined lattice parameters (a, b, c) decrease monotonically with In doping, leading to an almost linear unit‐cell volume contraction of ∼0.2% as x increases from 0 to 0.0832 (Table S2). This Vegard‐like trend indicates substitutional incorporation of the smaller In^3^^+^ ions on Ag^+^ sites. The anisotropic shrinkage, with a slightly larger reduction along the c‐axis, suggests subtle distortions in the local coordination environment, likely arising from the aliovalent nature of In^3^^+^ doping and associated charge‐compensation mechanisms. Such lattice contraction is expected to influence the electronic bandwidth and phonon dispersion, thereby impacting transport properties. Moreover, scanning electron microscopy (SEM) images (Figure S2) reveal that In doping does not significantly alter the morphology or grain size of Ag_2_Se.

X‐ray photoelectron spectroscopy (XPS) results of Ag_1.9287_In_0.0713_Se are presented in Figure 1e–g. The In 3d spectrum exhibits two distinct peaks at 445.06 eV (In 3d_5/2_) and 452.56 eV (In 3d_3/2_), characteristic of In^3+^, confirming the successful incorporation of In rather than the formation of segregated In─Se phases [42]. After doping, the Se 3d peaks shift noticeably toward lower binding energies, whereas the Ag 3d_5/2_ and Ag 3d_3/2_ peaks remain nearly unchanged at ∼368.3 and ∼374.3 eV, consistent with Ag^+^. The slight downshift of the Se 3d binding energy suggests a modification of the local chemical environment of Se atoms upon In incorporation. Once incorporated into the lattice, In^3+^ may partially substitute Ag^+^, donating additional electrons to the neighboring Se^2−^ and thereby increasing the local electron cloud density, which reduces the binding energy [43, 44]. Furthermore, In doping may induce local lattice distortions that modify the coordination environment and charge distribution around Se, further enhancing the covalent character of the bond between Se and surrounding atoms [45]. These binding energy shifts provide clear evidence of the effective regulation of the electronic structure by In doping and indirectly verify the successful realization of substitutional doping.

**FIGURE 2:**
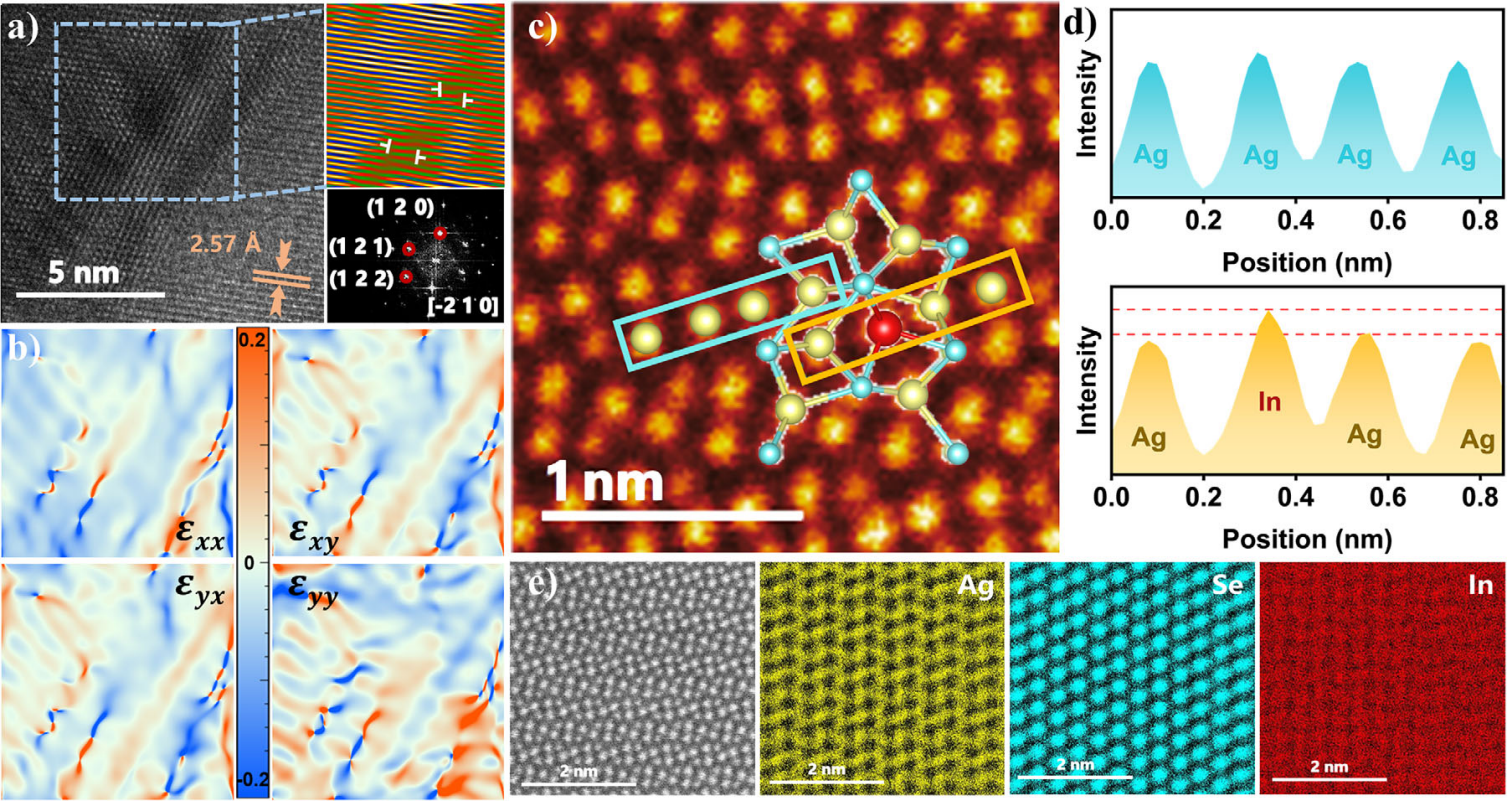
(a) HRTEM of Ag_1.9287_In_0.0713_Se, with the IFFT from the selected region on the upper right and the FFT pattern on the bottom right. (b) GPA of HRTEM image presented on the panel a. (c) HAADF‐STEM of Ag_1.9287_In_0.0713_Se viewed along [100] zone axis. (d) Intensity images of atoms marked with rectangular frames in panel c. (e) EDX elemental mapping depends on HAADF‐STEM of Ag_1.9287_In_0.0713_Se.

High‐resolution transmission electron microscopy (HRTEM) images of In‐doped Ag_2_Se (Figure 2a) display well‐resolved lattice fringes with a spacing of ∼2.57 Å, consistent with the (121) planes. Compared with the pristine sample (Figure S3), the local lattice exhibits pronounced bending and discontinuities, indicative of a high density of dislocations and associated strain fields. The fast Fourier transform (FFT) pattern shows three prominent adjacent diffraction spots corresponding to the (120), (121), and (200) planes. The inverse FFT (IFFT) from a selected region highlights the dislocation network, while the corresponding FFT pattern confirms retention of the orthorhombic phase, accompanied by weak extra reflections that suggest local symmetry breaking and periodic lattice modulation induced by In doping. To further probe lattice distortions, geometric phase analysis (GPA) was performed on the HRTEM images (Figure 2b). The GPA maps reveal alternating tensile and compressive regions in the *ε_xx_* and *ε_yy_* components, along with pronounced shear strain (*ε_xy_*, *ε_yx_*) localized at dislocation cores. The spatial correlation between these high‐strain regions and the dislocation network underscores the anisotropic nature of the lattice deformation. Such local strain fields, together with doping‐induced dislocations, are expected to act as efficient scattering centers for mid‐ to high‐frequency phonons, thereby contributing to the substantial reduction of lattice thermal conductivity [46].

The Ag and Se sublattices can be unambiguously resolved in the atomic‐resolution HAADF‐STEM image of Ag_2_Se along the [100] zone axis (Figure 2c). Ideally, six Ag atoms are expected to occupy the positions within a “hexagon” formed by six Se atoms, with an additional Se atom located at the hexagon center [47]. However, comparison of the atomic intensities among four parallel Ag sites reveals that the red‐marked atoms exhibit higher contrast than the others (Figure 2d). Given that the atomic numbers of In and Ag differ by only two, the STEM contrast variation is subtle. Nonetheless, this intensity difference suggests that Ag atoms are partially substituted by In at the tetracoordinate sites. Such substitution is likely to introduce both mass and size mismatch, thereby perturbing the local coordination environment. A larger‐area atomic‐resolution STEM‐HAADF image has been exhibited in Figure S4a, which allows evaluation of contrast variations over a statistically meaningful region rather than relying on isolated atomic columns. Within this field of view, eight atomic columns exhibit systematically enhanced HAADF intensity compared with surrounding Ag columns. Line‐profile analyses extracted across these columns consistently show a higher scattering intensity, suggesting the presence of heavier atomic species relative to Ag. Importantly, these features are spatially isolated and sparsely distributed, rather than forming contiguous regions. Energy‐dispersive X‐ray spectroscopy (EDX) elemental mapping (Figure 2e) further supports this interpretation: Ag and Se show a continuous lattice distribution, while weak but discernible In signals are co‐localized with specific Ag columns. Figure S4b,c shows the electron energy loss spectroscopy (EELS) of Ag_1.9287_In_0.0713_Se. Due to the doping concentration of In and the dominance of Ag in the host lattice, the In‐related EELS signal in Figure S4b is expected to be weak and partially overlapped with the Ag M_4,5_ edges [48]. Nevertheless, the absence of sharp or isolated In‐related edges characteristic of In‐rich secondary phases suggests that In may be highly dispersed within the Ag‐based lattice, rather than forming segregated clusters. With the Se L_2,3_ edges exhibit no additional features indicative of secondary In─Se phases [49, 50]. The absence of spectral signatures associated with In─Se‐rich compounds, together with the homogeneous EDS mapping, supports a substitutional rather than segregated incorporation mechanism. SEM‐EDX elemental maps also indicate that the nanocrystals of Ag_2‐x_In_x_Se are composed of Ag, In, and Se, with all elements distributed uniformly throughout the sample (Figures S5 and S6). The Ag_2‐x_In_x_Se nanocrystals were then vacuum‐dried and hot‐pressed into pellets to characterize their thermoelectric properties (Table S3). XRD patterns of Ag_2‐x_In_x_Se powder after annealing and pellets were illustrated in Figures S7 and S8.

**FIGURE 3:**
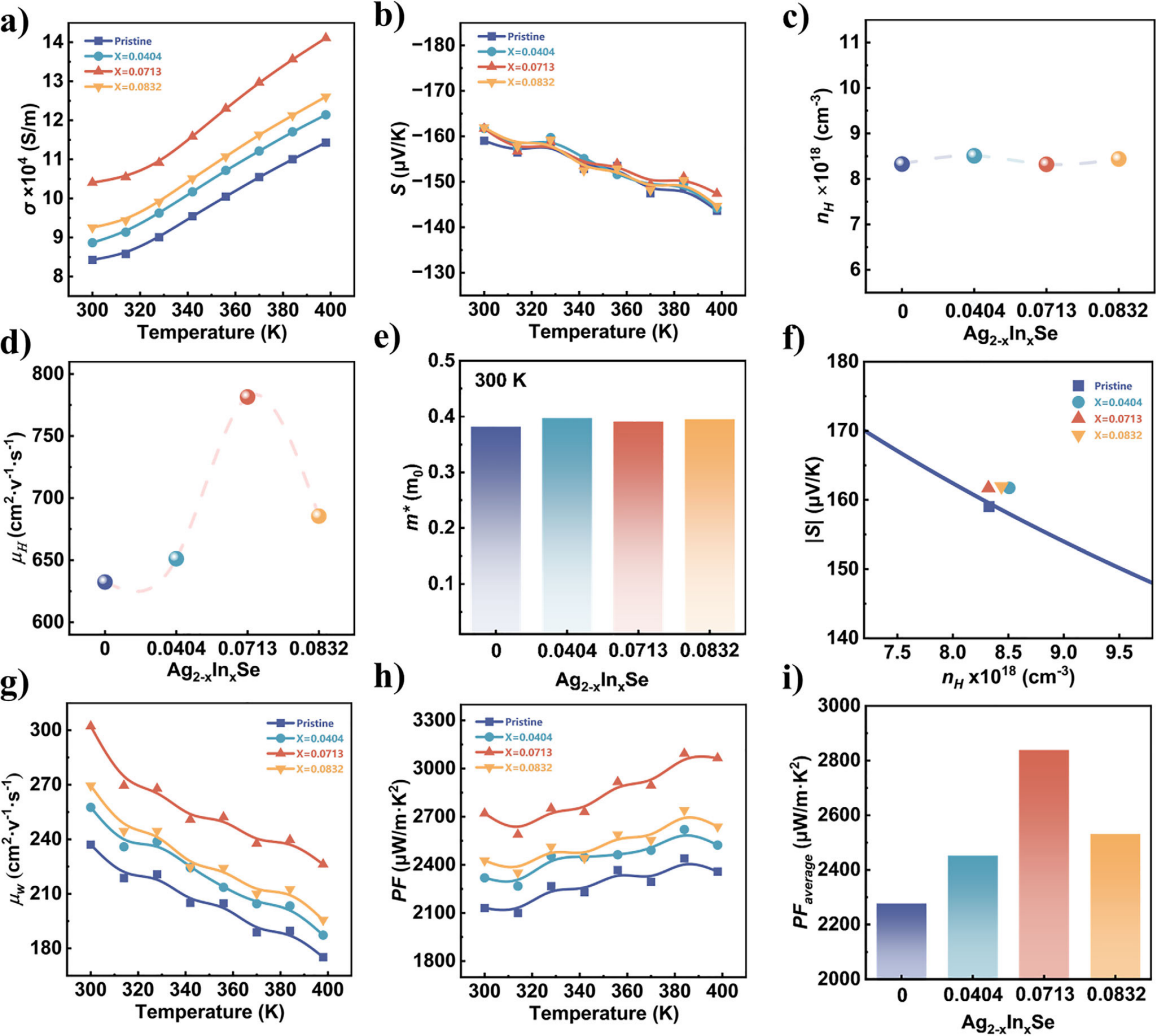
(a–i) Thermoelectric properties of Ag_2‐x_In_x_Se pellets. (a) Temperature dependence of *𝜎*. (b) Temperature dependence of *S*. (c) *n_H_* and (d) *µ_H_* of Ag_2‐x_In_x_Se under room temperature. (e) m^*^. (f) Pisarenko plot. (g) Temperature dependence of *µ_w_*. (h) *PF*. (i) Average *PF*.

The incorporation of indium into Ag_2_Se induces pronounced modifications in both the microstructure and charge transport, enabling a concerted optimization of electrical and thermal properties. As shown in Figure 3a, In‐doped samples exhibit significantly higher electrical conductivity (*σ*) across the entire temperature range compared to pristine Ag_2_Se. In contrast, the Seebeck coefficient (*S*) remains nearly unchanged with doping, with the curves for different concentrations largely overlapping (Figure 3b). This indicates that the enhanced *σ* does not arise from substantial changes in carrier concentration. Hall measurements at room temperature confirm this interpretation: the carrier concentration (*n_H_*) varies only slightly with doping, whereas the carrier mobility (*µ_H_*) shows a pronounced enhancement, peaking at x = 0.0713 before slightly declining at higher concentrations (Figure 3c,d). The effective mass (m^*^) exhibits only a minor increase with In substitution (Figure 3e), which helps sustain or slightly improve *S*, consistent with the Pisarenko relation shown in Figure 3f. Notably, the Seebeck coefficients of In‐doped samples systematically lie above the single parabolic band (SPB) prediction for pristine Ag_2_Se, further corroborating the modest enhancement in m^*^. Moreover, the weighted mobility (*µ_w_*), a key descriptor of electronic transport quality, is significantly elevated upon In incorporation (Figure 3g), reflecting suppressed carrier scattering. The concurrent enhancement of these two mobilities indicates that the improvement in carrier transport is intrinsic in nature, rather than arising from changes in carrier concentration. Such mobility enhancement can be attributed to reduced carrier scattering and a more favorable transport environment induced by In incorporation, which is consistent with the reduced activation energy extracted from the temperature‐dependent conductivity.

Consequently, a favorable balance between conductivity and Seebeck coefficient is achieved, yielding a substantially improved power factor (*PF*) of ∼3100 µW m^−^^1^ K^−^^2^ at 398 K, with the average PF also markedly enhanced to 2838 µW m^−^^1^ K^−^^2^ (Figure 3h,i). Thermoelectric measurements performed along directions parallel and perpendicular to the pressing direction show nearly identical transport behavior (Figure S9), suggesting negligible directional dependence.

**FIGURE 4:**
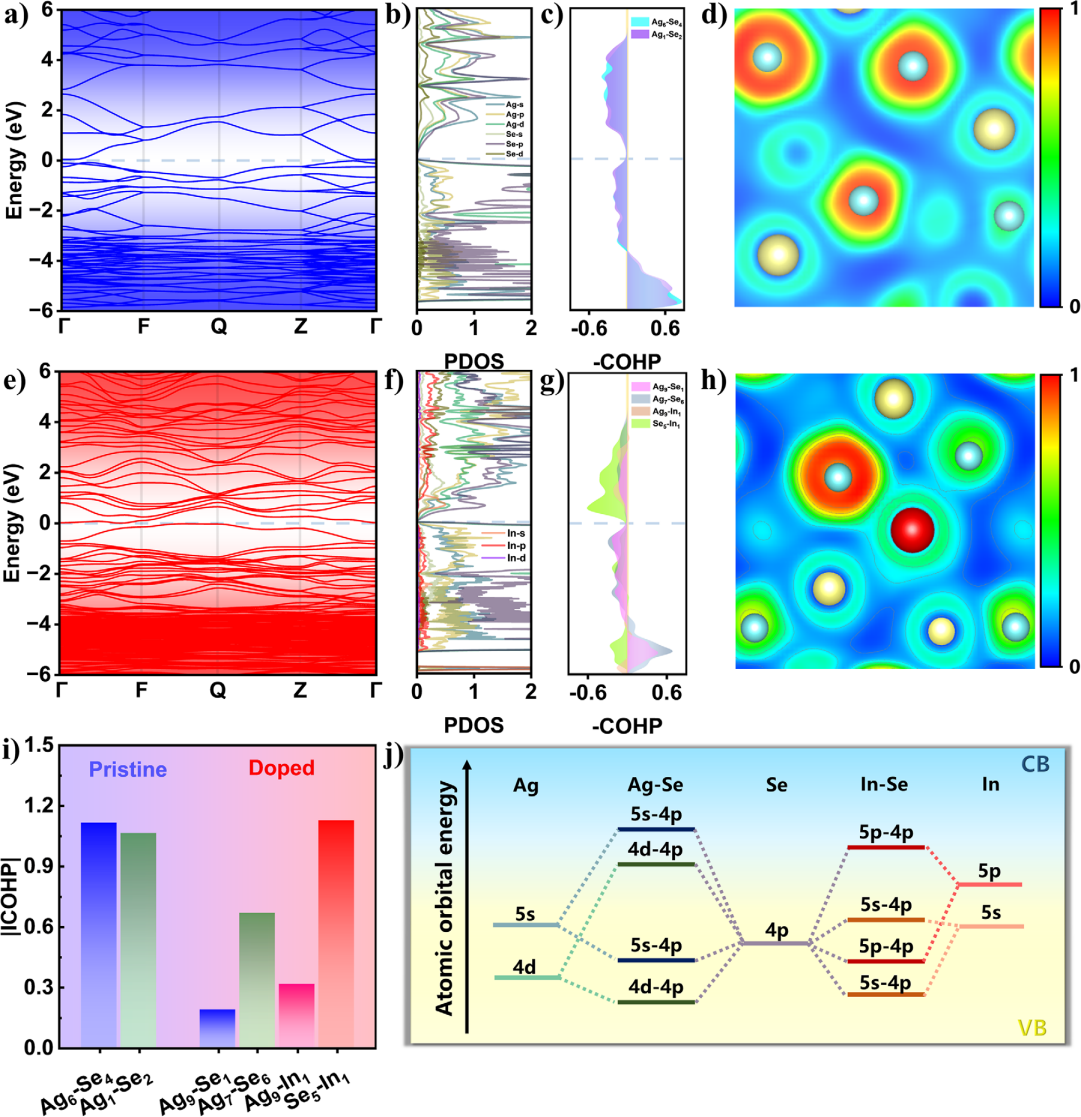
(a) Band structure; (b) Projected density of state; (c) Crystal orbital Hamilton population; d) Electron localization function of Ag_2_Se. (e–h) Band structure, projected density of state, Crystal Orbital Hamilton population, and Electron Localization Function of Ag_1.9287_In_0.0713_Se. (i) Absolute values of ICOHP before and after In doping. (j) Schematic atomic orbital hybridization for Ag─Se and In─Se bonding.

To elucidate the influence of In doping on the electronic band structure of Ag_2_Se, first‐principles calculations were performed to reveal the intrinsic electronic effects induced by In substitution (Figure S10). For pristine Ag_2_Se (Figure 4a–d), the band structure exhibits a narrow indirect gap of 0.048 eV, consistent with its semi‐metallic behavior near room temperature [51]. The projected density of states (PDOS) shows that the valence band maximum (VBM) is primarily composed of Se 4p and Ag 4d orbitals, while the conduction band minimum (CBM) is dominated by Ag 5s, Ag 4d, and Se 4p orbitals, reflecting the strong Ag─Se hybridization.

Upon In doping, the band gap further narrows to 0.027 eV, arising from a slight upward shift of the VBM and a downward shift of the CBM (Figure 4e–h). This band‐gap reduction is accompanied by a redistribution of orbital contributions in the PDOS, where In 5s and 5p states emerge near the Fermi level, weakening the original Ag s‐d hybridization. Compared with the pristine case (Figure 4c), the Crystal Orbital Hamilton Population (COHP) analysis (Figure 4g) indicates reduced antibonding strength for both tricoordinate and tetracoordinate Ag sites near the Fermi level, consistent with a local structural distortion induced by In substitution at the tetracoordinate site. These bonding modifications originate from the larger atomic radius and distinct valence configuration of In, which introduce anisotropic local strain fields and alter charge distribution.

The Integrated COHP (ICOHP) provides a quantitative measure of total bond strength by summing orbital interactions within a given energy window [52]. For the intrinsic structure, the covalent bond strength between Ag atoms in a tricoordinated configuration is higher than that between Ag atoms in a tetracoordinated configuration. After In doping with In atoms substituting Ag sites, the system undergoes local structural distortion. The doped In atoms compete for electrons with adjacent Ag atoms, leading to the attenuation of the Ag─Se covalent bond strength. In addition, the Se‐In bonds feature shorter bond lengths and higher orbital overlap, resulting in their ICOHP values being significantly larger than those of Ag‐In bonds (Figure 4i). This observation is corroborated by the Electron Localization Function (ELF, Figure 4d,h), which reveals enhanced charge localization around In and adjacent Se atoms, in agreement with the XPS results. A schematic illustration of orbital hybridization (Figure 4j) highlights the difference between Ag─Se and In─Se bonding. According to valence bond theory, Ag atoms adopt weak s‐d hybridization to stabilize a tetrahedral coordination, while tricoordinate sites accommodate a lone electron pair in an s‐d hybrid orbital, forming a trigonal pyramid [53, 54]. When In selectively substitutes tetracoordinate Ag, the superior orbital overlap and shorter bond length of the In─Se bond reinforce local covalency and suppress band broadening through bond‐network reconstruction, thereby enhancing carrier mobility and electrical conductivity. Furthermore, to verify the structural stability of the two systems, we evaluated the thermodynamic stability of Ag_2_Se and In doped Ag_2_Se at 300 K via ab initio molecular dynamics (AIMD) simulations, with the corresponding results displayed in Figures S11 and S12. During the total simulation duration of 4 ps, the free energies of both systems fluctuate within a narrow range without any bond breakage, indicating that they can maintain excellent thermal stability at this temperature.

Overall, the band structure comparison indicates that the Fermi level shifts deeper into the conduction band after In substitution, confirming its effective donor role and the associated increase in carrier concentration, which primarily accounts for the improved electrical conductivity.

Importantly, the conduction band dispersion near the band edge remains largely preserved, and no flat impurity bands or strongly localized states are introduced. The projected density of states (Figure 4f) reveals significant hybridization between In 5p and Se 4p orbitals, indicating that In contributes to the extended conduction framework rather than forming localized trap states. This interpretation is further supported by the charge density distribution (Figure 4h), which shows delocalized electronic states without pronounced localization around the In sites.

Although doping can introduce impurity scattering, the absence of localized electronic states and the maintained band dispersion help prevent severe mobility degradation. In addition, the increased carrier concentration enhances electrostatic screening of ionized impurity potentials, thereby reducing scattering strength. Consequently, mobility is not suppressed and even moderately enhanced, resulting in the observed increase in both Hall mobility and weighted mobility.

**FIGURE 5:**
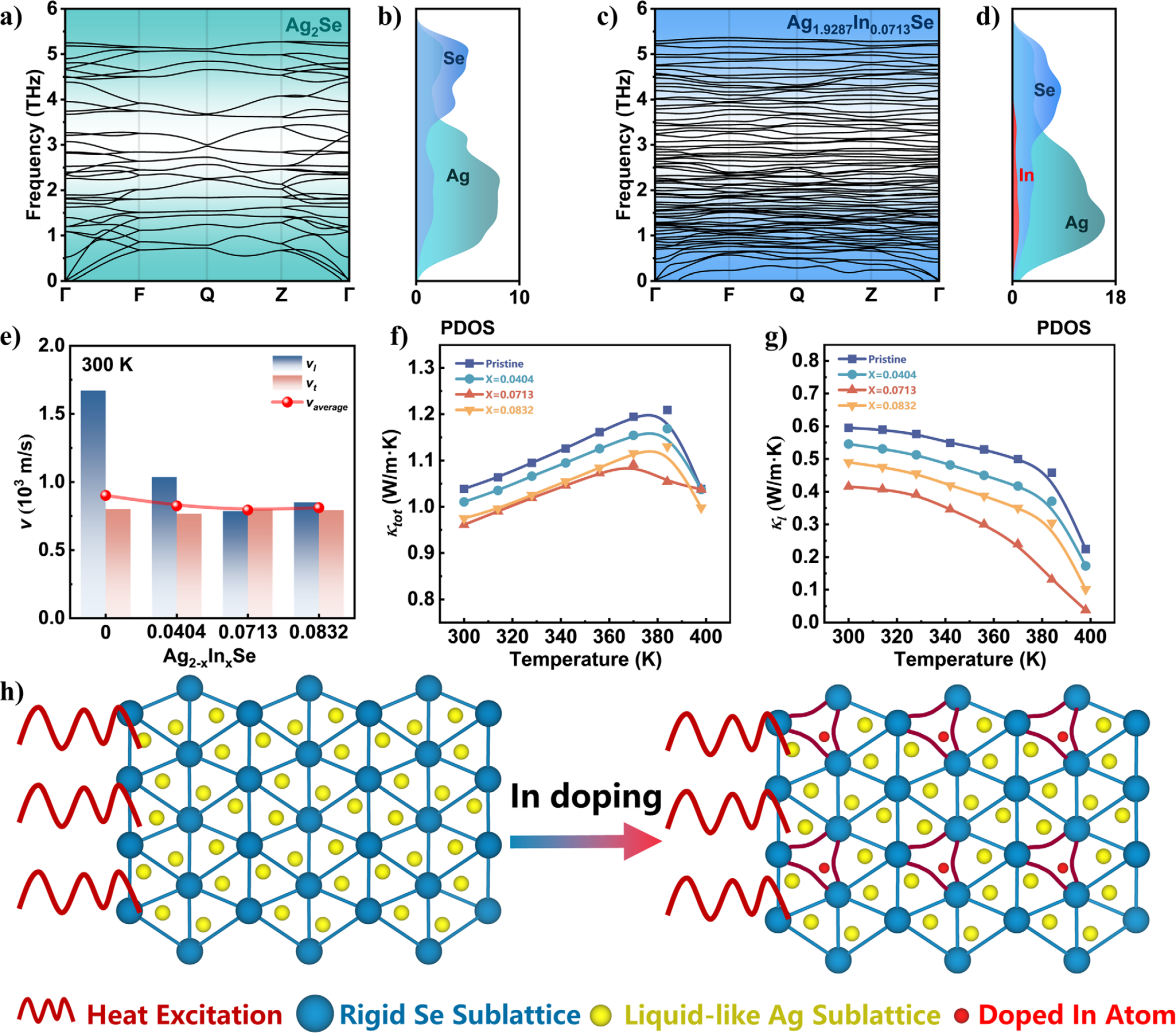
(a) Phonon dispersion curves and (b) Projected DOS of pristine Ag_2_Se. (c,d) Phonon dispersion curves and Projected DOS of Ag_1.9287_In_0.0713_Se. (e) Acoustic sound velocity of Ag_2‐x_In_x_Se at room temperature. (f) *κ_tot_* and (g) *κ*_*l*_ of the Ag_2‐x_In_x_Se pellets. (h) Schematic illustration of the trade‐off between electronic and phononic transport channels in moderately In‐doped Ag_2_Se.

The pristine phonon spectrum (Figure 5a,b) exhibits well‐separated branches, with strongly dispersive acoustic modes near Γ and distinct Ag─Se contributions in the phonon DOS. Upon In substitution, the phonon bands become markedly flattened (Figure 5c), particularly within 0–2 THz, accompanied by a substantial increase in low‐frequency DOS. Moreover, In‐induced localized vibrations introduce extra mid/low‐frequency contributions to the PDOS (Figure 5d). These features‐enhanced low‐frequency DOS and increased vibrational localization are clear signatures of lattice softening, which suppresses lattice thermal transport by reducing the contribution of long‐wavelength heat‐carrying phonons [55]. The electronic thermal conductivity (*κ_e_*) increases only slightly due to the higher *σ* (Figure S13), leaving the lattice contribution (*κ*_*l*_) as the dominant factor governing the total thermal conductivity. At 300 K, the average sound velocities of the doped samples (Figure 5e; Table S4) exhibit a slight reduction, providing additional evidence of lattice softening induced by In doping, consistent with first‐principles calculations.

As shown in Figure 5f, *κ_tot_* exhibits a non‐monotonic temperature dependence, increasing at lower temperatures and then decreasing sharply above ∼380 K. Notably, *κ*_*l*_ of In doped samples are significantly suppressed across the entire temperature range compared with the pristine (Figure 5g), reaching below 0.1 W m^−^^1^ K^−^^1^ at 398 K for the moderately doped sample (x = 0.0713). To ensure reproducibility, three additional samples with the same doping concentration were prepared and measured for thermal conductivity; the results showed that *κ*_*l*_ at 398 K remained close to below 0.1 W m^−^^1^ K^−^^1^ in Figure S14a,b. This behavior originates from the competition between *κ*_*l*_ and *κ_e_*. At relatively low temperatures, the increase in *κ_tot_* is primarily attributed to the enhanced *κ_e_*. As temperature increases, *κ*_*l*_ becomes strongly suppressed due to intensified phonon scattering and the thermally activated disorder of the Ag sublattice, a characteristic feature of Ag_2_Se. Above ∼380 K, the rapid reduction in *κ*_*l*_ outweighs the electronic contribution, leading to the observed sharp decline in *κ_tot_*. To further validate the thermal transport behavior, time‐domain thermoreflectance (TTR) measurements were conducted as an independent experimental probe of thermal conductivity at 300 K (Figure S14c). The results reveal a trend consistent with that observed in Figure 5f, while the extracted thermal conductivity values are slightly lower.

In addition, to clarify the microscopic origin of the reduced lattice thermal conductivity, we have further analyzed the phonon properties based on the calculated mode‐resolved Grüneisen parameters and phonon relaxation times in Figure S15. In doping significantly enlarges the mode‐resolved Grüneisen constants, particularly in the low‐frequency acoustic branches. The increased Grüneisen parameters indicate enhanced lattice anharmonicity, which strengthens three‐phonon scattering processes. Correspondingly, the calculated phonon relaxation times are substantially reduced after doping, especially for low‐frequency acoustic phonons that dominate lattice heat transport. Because lattice thermal conductivity is primarily governed by long‐wavelength acoustic phonons with large group velocities and long lifetimes, the pronounced reduction in their relaxation times inevitably leads to a strong suppression of *κ*_*l*_. These results indicate that the reduction in *κ*_*l*_ arise from multiscale phonon scattering combined with lattice softening. High‐temperature XRD (Figure S16) confirms that this sample maintains the low‐temperature orthorhombic phase of Ag_2_Se throughout the temperature range, while differential scanning calorimetry (DSC) analysis (Figure S17) reveals a phase transition temperature of 407 K for all compositions.

Benefiting from the unique phonon‐liquid electron‐crystal (PLEC) nature of Ag_2_Se, partial substitution of tetracoordinate Ag by In yields synergistic optimization in both electronic and phononic transport. On the electronic side, In─Se bonds provide improved orbital overlap compared to Ag─Se bonds, enhancing local covalency and suppressing band broadening, which in turn increases carrier mobility in the phonon‐scattering‐dominated regime. Enhanced lattice stiffness further mitigates electron scattering, contributing to higher conductivity (Figure 5h). On the phononic side, In doping introduces strong mass‐difference scattering and local strain‐field scattering, which, together with lattice softening and the intrinsically low lattice thermal conductivity, substantially shorten phonon mean free paths and further reduce *κ_l_*. Importantly, because electronic transport in Ag_2_Se mainly occurs through the stable Se sublattice, these additional scattering mechanisms exert only a minor influence on carrier mobility. However, excessive In incorporation reverses these benefits: while In─Se bonds strengthen covalency, the proliferation of impurity scattering centers and long‐range lattice distortions drastically shorten carrier mean free paths, increase effective mass, and may even disrupt band continuity, ultimately reducing carrier mobility.

**FIGURE 6:**
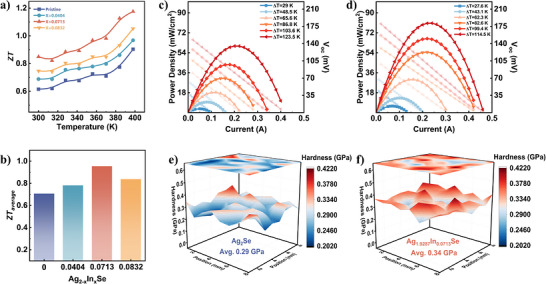
(a) *ZT* value and (b) *ZT_avg_* value of the Ag_2‐x_In_x_Se pellets. Power density and open‐circuit voltage of multi‐leg devices using (c) pristine Ag_2_Se and (d) Ag_1.9287_In_0.0713_Se as n‐type thermoelectric legs. (e,f) 3D cloud diagram of hardness for Ag_2_Se and Ag_1.9287_In_0.0713_Se.

Therefore, moderate In substitution at tetracoordinate Ag sites establishes an optimal balance between electronic and phononic transport, markedly suppressing *κ_l_* while enhancing electrical conductivity. This dual optimization enables a synergistic boost in thermoelectric performance, yielding a peak *ZT* of ∼1.2 at 398 K and an average *ZT* of 0.96 for Ag_1.9287_In_0.0713_Se samples. (Figure 6a,b).

To assess the practical applicability of In‐substituted Ag_2_Se, we fabricated multi‐leg thermoelectric modules using pristine Ag_2_Se and the optimized In‐doped composition (x = 0.0713) as n‐type legs after electroplating Ni layer (Figure S18), paired with commercial Bi_0.5_Sb_1.5_Te_3_ p‐type legs. Key parameters of multi‐leg devices were exhibited in Table S5. The output power density‐current and voltage‐current characteristics under various temperature gradients (ΔT) demonstrate that the In‐doped module consistently achieves higher open‐circuit voltages and greater power density than its pristine counterpart across the entire ΔT range (Figure 6c,d). This improvement arises directly from the enhanced *ZT* of the doped n‐type leg, resulting from the concurrent suppression of lattice thermal conductivity and enhancement of carrier mobility, which contributes to the boost in energy conversion efficiency (Figure S19). The superior module‐level performance highlights the effectiveness of the proposed microstructural and electronic optimization strategy, validating its impact not only at the material level but also in device‐relevant architectures. To evaluate operational stability, thermal cycling tests were conducted using a single‐leg device configuration to decouple intrinsic material stability from module‐level integration effects. This simplified geometry ensures a well‐defined thermal gradient and electrical bias condition, which is particularly critical for Ag_2_Se‐based systems with mobile Ag sublattices. The single‐leg device demonstrates highly reversible thermoelectric performance over 383 thermal cycles, maintaining a stable output voltage of 5.5 mV under a well‐confined temperature gradient. No observable temporal degradation or spatial failure features are detected, indicating robust operational stability under coupled thermal and electrical stress (Figure S20).

Nanoindentation measurements were conducted to evaluate the mechanical properties of the samples, and the corresponding hardness maps are presented in Figure 6e,f [56]. The pristine Ag_2_Se exhibits an average hardness of 0.29 GPa, whereas the optimized In‐doped sample reaches 0.33 GPa and displays a more homogeneous spatial distribution of hardness. In addition, the In‐substituted sample shows a smaller indentation size (Figure S21), indicating enhanced resistance to localized plastic deformation. This enhancement is ascribed to lattice stiffening induced by local strain fields and improved grain boundary cohesion upon In incorporation, which partially mitigates the mechanical anisotropy of the liquid‐like Ag sublattice while concurrently contributing to *κ_l_* reduction. Taken together, these findings establish that moderate In substitution enables a rare combination of superior thermoelectric efficiency and enhanced mechanical stability, underscoring its promise for practical device applications.

## 3 Conclusion

In this work, we developed a facile wet‐chemical route to synthesize orthorhombic Ag_2_Se and In‐substituted Ag_2_Se nanocrystals with controlled dopant site occupancy. In preferentially replaces tetracoordinate Ag atoms, introducing local strain fields, dislocation networks, and mass‐size mismatch defects while preserving the orthorhombic framework. These microstructural perturbations act as efficient phonon scatterers, yielding ultralow lattice thermal conductivity (<0.1 W m^−^^1^ K^−^^1^ at 398 K). First‐principles calculations reveal that In─Se bonds provide superior orbital matching and weaken Ag─Se antibonding, narrowing the band gap, reducing potential barriers, and enhancing carrier mobility. The combined electronic and phononic optimization results in a peak *ZT* of ∼1.2 at moderate doping. Module‐level tests confirm that the optimized n‐type leg consistently outperforms the pristine counterpart, while hardness mapping indicates improved mechanical robustness due to lattice stiffening. These findings position In‐substituted Ag_2_Se as a promising n‐type thermoelectric with tunable transport channels, offering a practical pathway for integrating PLEC materials into high‐performance, mechanically reliable devices.

## Conflicts of Interest

The authors declare no conflicts of interest.

## Supporting information




**Supporting File**: advs75408‐sup‐0001‐SuppMat.docx.

## Data Availability

The data that support the findings of this study are available from the corresponding author upon reasonable request.
