# Supplementary material for: Defect Driven Electronic Structure Reconfiguration and Hierarchical Phonon Suppression in Ag2Se Thermoelectrics
Source: Adv Sci (Weinh). 2026 Apr 21;13(40):e75408. doi: 10.1002/advs.75408 (PMC13335593; doi:10.1002/advs.75408)
Supplement: Supplementary file 1 — Supporting File: advs75408‐sup‐0001‐SuppMat.docx. [file ADVS-13-e75408-s001.docx]

Supplementary Information

**Defect Driven Electronic Structure Reconfiguration and**

**Hierarchical Phonon Suppression in Ag_2_Se Thermoelectrics**

Yineng Gou^1&3^, Mengyao Li^2^*, Fang Lyu^1^, Xiaolong Sun^1^, Muyang Huang^3^, Wenhao Xie^3^, Liangwei Hu^3^, Wei Cao^3^, Yue Hou^3^, Yong Liu^1&4^, Andreu Cabot^5&6^, Ziyu Wang^1&4^*

^1^ School of Physics and Technology, Wuhan University, Wuhan, 430072, China.

^2^ School of Physics and Laboratory of Zhongyuan Light, Zhengzhou University, Zhengzhou 450001, China.

^3^ School of Integrated Circuits, Wuhan University, Wuhan, 430072, China.

^4^ Key Laboratory of Artificial Micro-and Nano-structures of Ministry of Education and School of Physics and Technology, Wuhan University, Wuhan, 430072, China.

^5^ Catalonia Institute for Energy Research-IREC, Sant Adrià de Besòs, 08930 Barcelona, Spain.

^6^ ICREA, Pg. Lluís Companys 23, 08010 Barcelona, Catalonia, Spain.

*Corresponding author: [limengyaorz@zzu.edu.cn](mailto:limengyaorz@zzu.edu.cn); [zywang@whu.edu.cn](mailto:zywang@whu.edu.cn)

**Materials and Methods**

***Chemicals***

Selenium powder (Se, >99.9%), ethylene glycol (EG, AR), and Indium nitrate trihydrate (In(NO_3_)_3_·xH_2_O, >99.99%) were purchased from Aladdin, China. Silver nitrite (AgNO_3_), acetone (C_3_H_6_O), and ethyl alcohol (C_2_H_6_O) were purchased from Sinopharm Chemical Reagent. All chemicals were used as received without further purification.

***Synthesis of Ag_2_Se nanocrystals***

The fabrication of pristine silver selenide nanocrystals was achieved using a facile wet chemical method. First, 180 mL of ethylene glycol and 17.7 mmol of selenium powder were added to a 250 mL two-neck flask, and the mixture was maintained at 323 K for 30 minutes to disperse the selenium powder in EG uniformly with glass stoppers, constructing an enclosed environment. Next, the flask was heated to 333 K, and 35.4 mmol of silver nitrate was added. The flask was then sealed with glass stoppers and left to stand for 4 hours. Once the mixture cooled to room temperature, it was transferred to a 50 mL centrifuge tube and centrifuged at 10,000 rpm for 5 minutes. The supernatant was transferred to a waste container, and the precipitate was sonicated separately in anhydrous ethanol and acetone, then centrifuged and rinsed to remove residual ethylene glycol and other organic byproducts. Finally, the sample was transferred to a vacuum drying oven and dried at 60°C for 12 hours to obtain silver selenide nanocrystals. The same process was used to prepare gradient In-doped samples. Add 3%, 5% and 7% In(NO_3_)_3_·xH_2_O based on AgNO_3_ to substitute Ag atoms with In atoms. The actual compositions are listed in Table S1.

***Bulk nanomaterial consolidation***

The dried powder was first calcined in a tubular furnace at 623 K for 1 hour. After the tube was cooled naturally to room temperature, the calcined powder was loaded into a 12.7 mm graphite die and processed via a spark plasma sintering (SPS) technique to gain a pellet. The SPS procedure was carried out at 423 K for 5 minutes, equipped with a sintering pressure of 70 MPa. The relative density of the pellets was measured by the Archimedes method (Table S2).

***Structural and chemical condition characterization***

X-ray diffraction (XRD) patterns were conducted on a Smartlab Studio II instrument (Rigaku Corporation, Japan) with Cu Kα radiation (λ = 1.5403 Å) at 40 keV and 40 mA. The morphology and energy dispersive spectrometer (EDS) [element analysis](https://www.sciencedirect.com/topics/materials-science/elemental-analysis) of the obtained samples were performed using [field emission scanning electron microscopy](https://www.sciencedirect.com/topics/materials-science/field-emission-scanning-electron-microscopy) (FESEM) with MIRA 3LMH and Zeiss SIGMA, instruments from England. High-resolution TEM (HRTEM) was carried out under the 200 keV Tecnai F20 field emission microscope, using an embedded Gatan quantum image filter. Scanning transmission electron microscopy with a high-angle annular dark-field detector (STEM-HAADF, JEM-ARM200CF, Japan) was employed to reveal the lattice structure. X-ray photoelectron spectroscopy (XPS, Thermo Escalab 250, USA) with a monochromatic Al K*α* X-ray source was used to characterize the elemental compositions of the sample surfaces. All binding energies were referenced to the C 1s peak (284.6 eV) arising from adventitious carbon. The elemental concentrations were analyzed using inductively coupled plasma optical emission spectroscopy (ICP-OES, Agilent 5110, USA). Standard solutions were used for calibration, and all samples were measured after acid digestion. Mechanical properties were evaluated using nanoindentation, measured by a Nano-Blitz 3D method (HXD-1000TMC/LCD).

***Thermoelectric Property Measurements***

Electrical parameters, including the Seebeck coefficient (*S*) and conductivity (*σ*), were simultaneously measured in a He atmosphere via a commercial apparatus (Cryoall CTA-3, China). The experimental errors of the *S* and *σ* measurements were ±5% and ±2%, respectively. The total thermal conductivity (*κ*) was calculated using actual bulk density (ρ), specific heat (*C_p_*), and thermal diffusivity (*D*) according to the equation *κ = C_p_ × D × ρ*, *C_p_, D* and *ρ* were measured using a Dulong-Petit limit instrument (Netzsch LFA-467, Germany), which employs both laser flash and Archimedes drainage methods. The specific heat capacity (*C_p_*) was measured using a differential scanning calorimeter (DSC, Switzerland). Additionally, the Hall coefficient (*R_H_*) of the specimens was assessed using van der Pauw’s method. The relationships between carrier concentration (*n_H_ = 1/(e·R_H_)*) and carrier mobility (*μ_H_ = σ·R_H_*) were then analyzed. These measurements were conducted at room temperature under a magnetic field of *H* = 0.6 T using a Lakeshore 7704 A Hall system.

***Performance Characterization of Multi-leg devices***

The multi-leg devices (1.06 cm × 1.06 cm × 2.6 mm) were measured as follows: tin paste was used to connect copper lines on both sides of the device, serving as electrodes. Voltage scanning measurements were then conducted using a Keithley 2450 digital source meter to obtain the *I-V* characteristic curve. One side of the device was placed on an electric hotplate, while the other was exposed to the air for natural cooling. The temperature of the hotplate was adjusted between each voltage scan to change the device's working conditions. A thermocouple was used to measure the temperatures of the hot and cold ends before each voltage scan.

***Estimation of the carrier effective mass m*:*** Effective masses (*m**) were estimated from the measured Seebeck coefficient and Hall carrier concentration (*n_H_*) using a single parabolic band (SPB) model with acoustic phonon scattering (*r* = -1/2). According to the following equations 1-3^[1-2]^.

$m^{*}={\frac{h^{2}}{2k_{B}}[\frac{n}{{4\pi F}_{1/2}}]}^{2/3}$ (1)

$S= \pm\frac{k_{B}}{e}\left( \frac{(r+3/2)F_{r+3/2}}{(r+3/2)F_{r+1/2}}-\eta\right)$ (2)

$F_{n}\left( \eta\right)=\int_{0}^{\infty} \frac{x^{n}}{1+e^{x-\eta}}dx$ (3)

where *η* is the reduced Fermi energy, *F_n_*(*η*) is the *n^th^*-order Fermi integral, *k_B_* is the Boltzmann constant, *e* is the electron charge, *h* is the Planck constant, and *r* is the scattering factor. The reduced Fermi energy was obtained by fitting the measured values of Seebeck coefficient.

***Sound velocity measurements***

The longitudinal (*v_l_*) and shear (*v_t_*) sound velocities were measured using an ultrasonic instrument (Ultrasonic Pulser/Receiver Model CTS-8077PR, SIUI, China) (Table S2). The average sound velocity (*v_avg_*) was calculated using the following equation^[3]^:

$v_{\mathrm{avg}}={[\frac{1}{3}(\frac{1}{v_{l}^{3}}+\frac{2}{v_{t}^{3}})]}^{-1/3}$ (4)

***Density Functional Theory calculations:***

Our first-principle calculations were carried out within the framework of density functional theory (DFT), as implemented in the Vienna ab initio Simulation Package (VASP)^[4]^. We employed the Perdew-Burke-Ernzerhof (PBE) generalized gradient approximation (GGA) for the exchange–correlation functional and used projector augmented-wave (PAW) pseudopotentials to describe the electron–ion interactions. The Brillouin zone was sampled with a plane-wave energy cutoff of 500 eV, ensuring a convergence of energy within 10⁻⁶ eV in self-consistent field calculations. Phonon dispersion relations and harmonic interatomic force constants were computed using the PHONOPY code^[5]^. For this purpose, 3×2×2 and 1×2×2 supercells were constructed for pure Ag_2_Se and In-doped Ag_2_Se, respectively. The projected crystal orbital Hamilton population (COHP) was analyzed using the LOBSTER package^[6]^. Importantly, for substitutional doping at the investigated concentration, the 1×2×2 supercell already captures the essential local coordination environment and electronic structure modulation around the In dopants. Previous studies^[7-8]^ have demonstrated that supercells of comparable size provide reliable and converged descriptions of electronic structure evolution in similar systems.


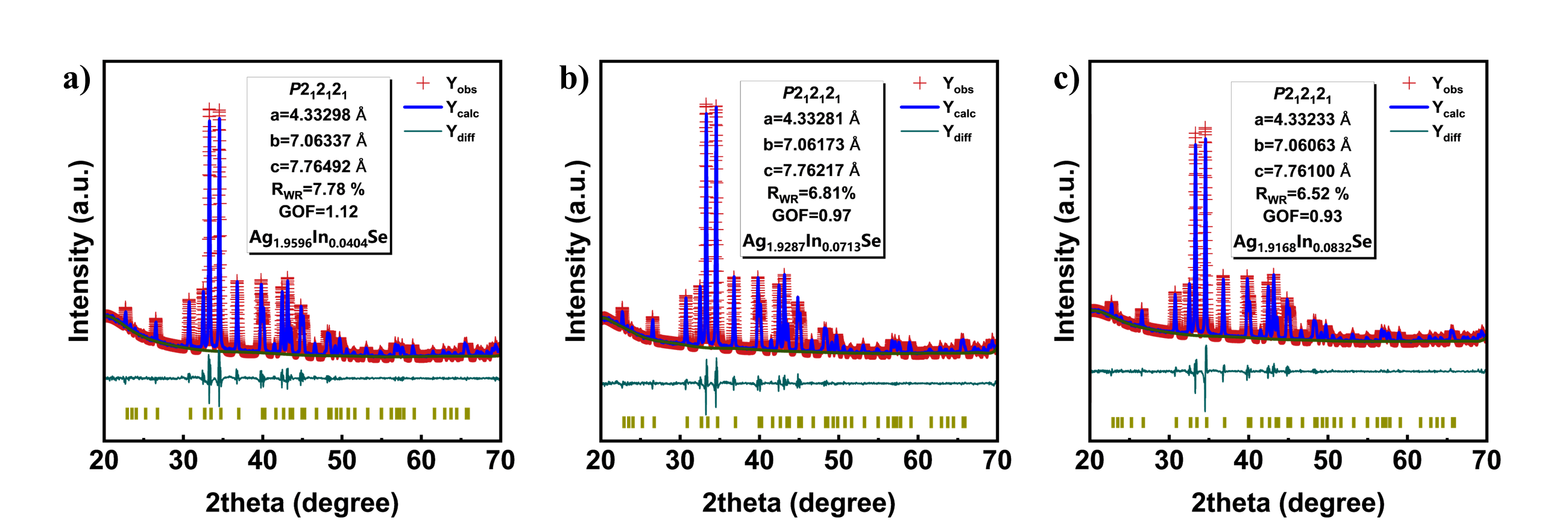


**Figure S1.** Rietveld refinement of the XRD pattern of Ag_2-x_In_x_Se samples.


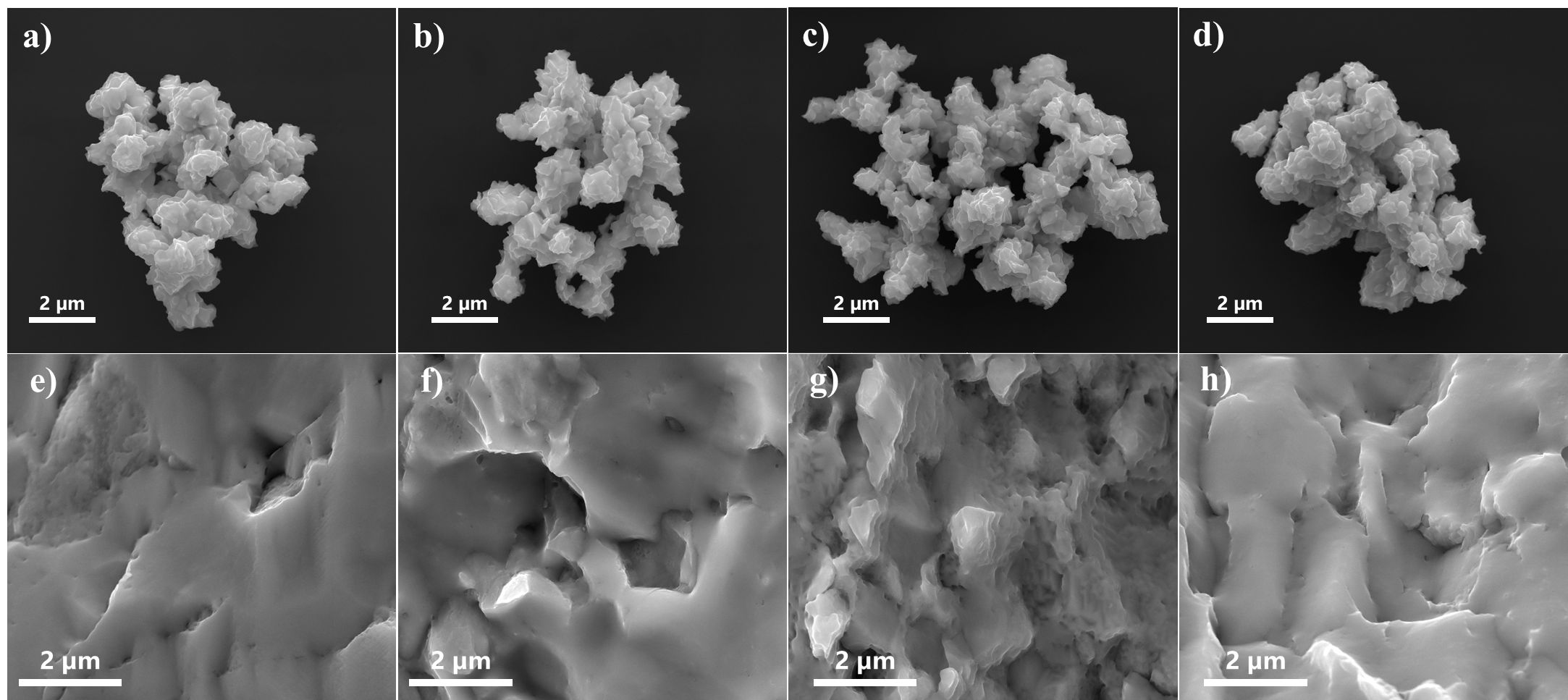


**Figure S2.** Scanning electron microscopy (SEM) images of a) pristine, b) Ag_1.9596_In_0.0404_Se, c) Ag_1.9287_In_0.0713_Se, d) Ag_1.9168_In_0.0832_Se nanocrystal; e-h) are the cross-sectional images of pellets corresponding to the frequence of SEM images.


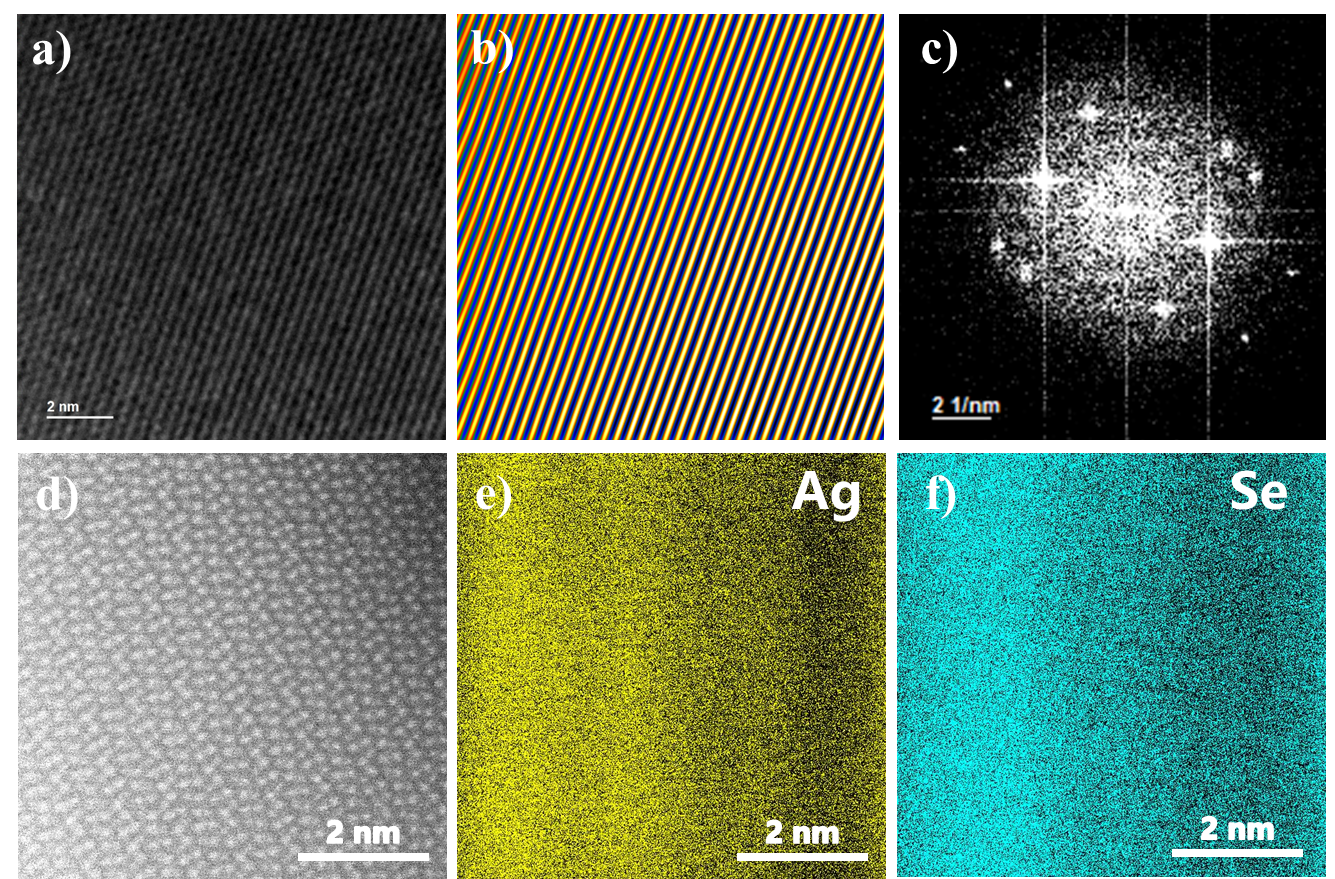


**Figure S3.** a) HRTEM, b) IFFT, c) FFT, d) STEM-HAADF and e-f) EDS mapping of Ag_2_Se pellet.


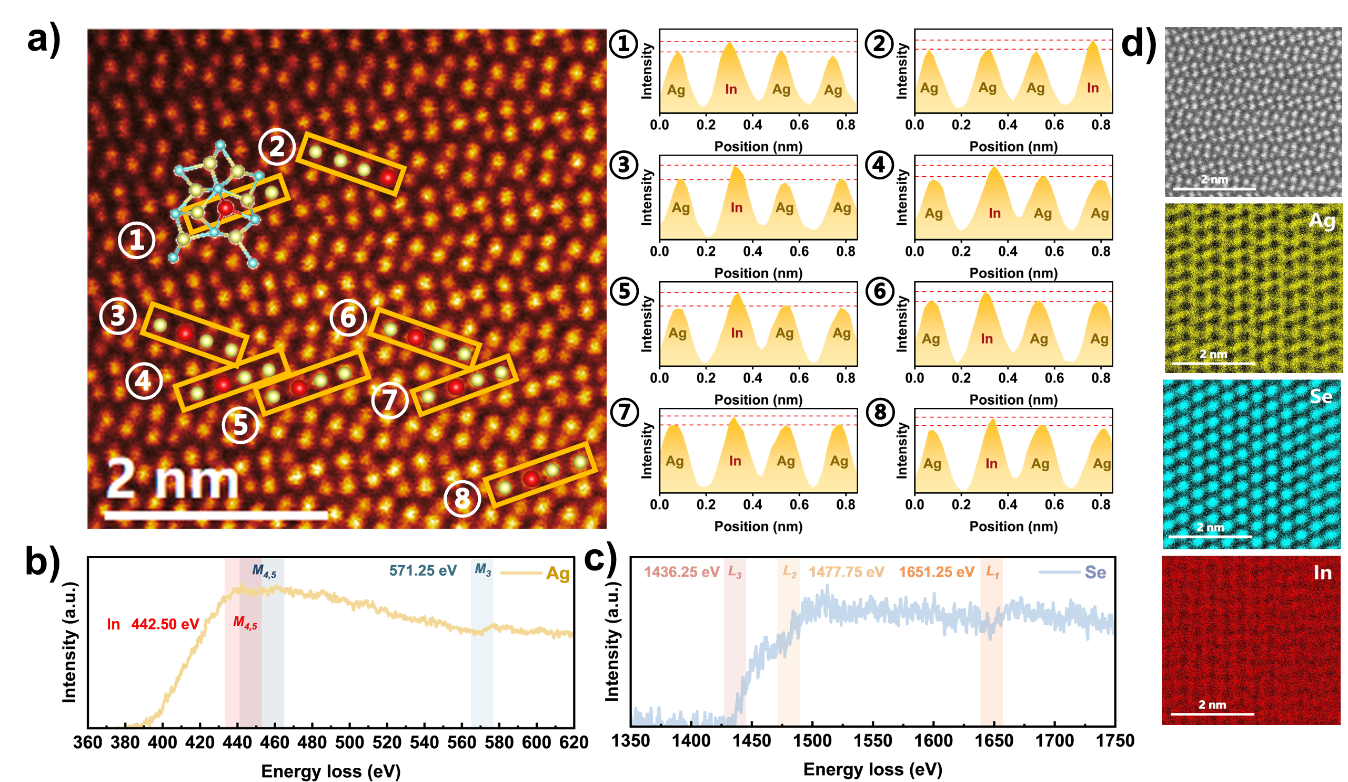


**Figure S4.** (a) Atomic-resolution STEM-HAADF of Ag_1.9287_In_0.0713_Se with statistical intensity analysis of atomic columns. Electron energy loss spectroscopy (EELS) of Ag_1.9287_In_0.0713_Se with (b) Ag and (c) Se. (d) EDX elemental mapping depends on STEM-HAADF of Ag_1.9287_In_0.0713_Se.


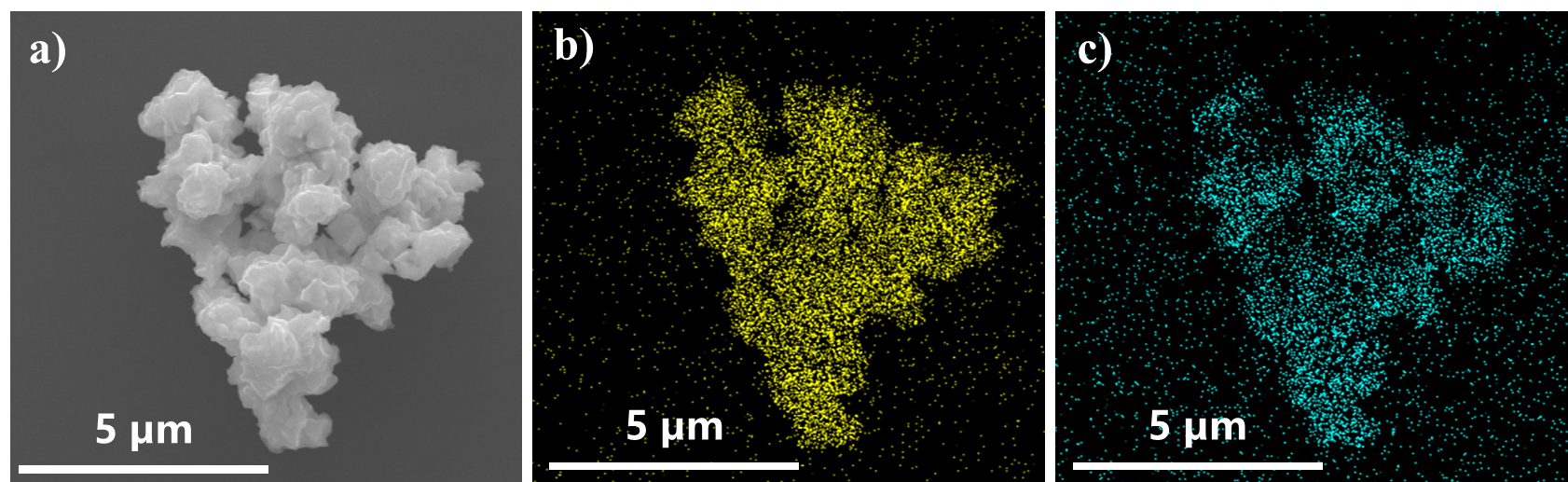


**Figure S5.** SEM images and EDS mappings of Ag_2_Se.


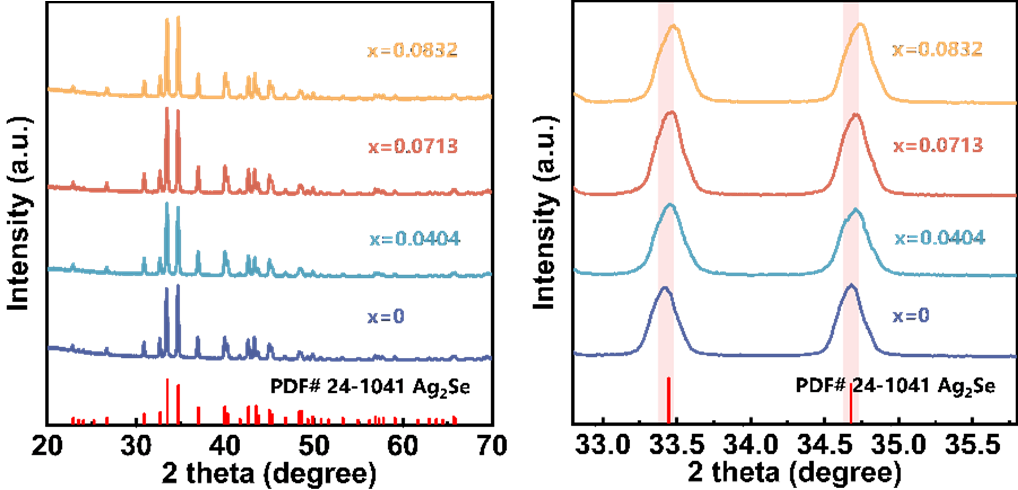

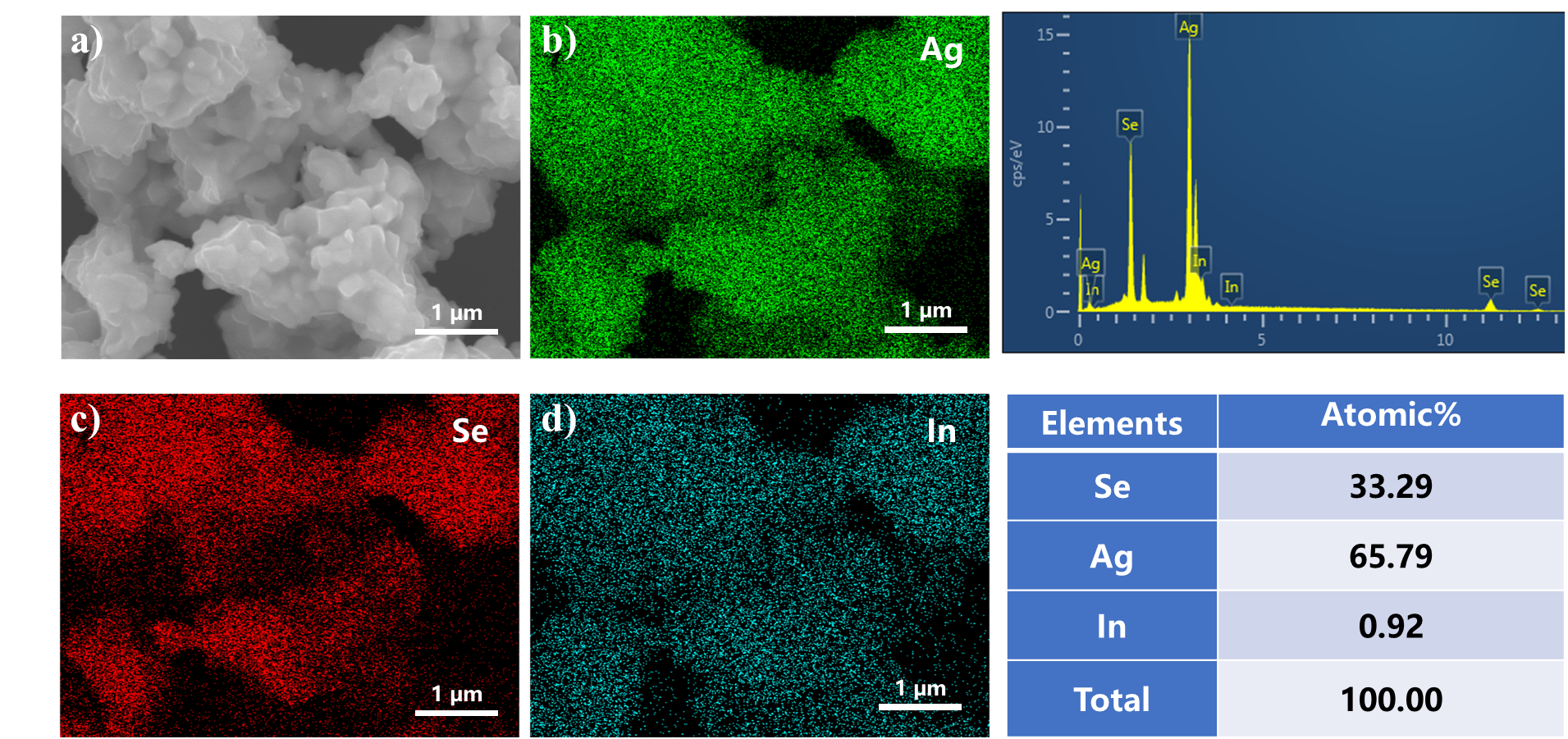
**Figure S6.** a) SEM images and b-d) EDS mappings of Ag_1.9287_In_0.0713_Se.

**Figure S7**. XRD patterns and localized enlarged image of Ag_2-x_In_x_Se after annealing.

*
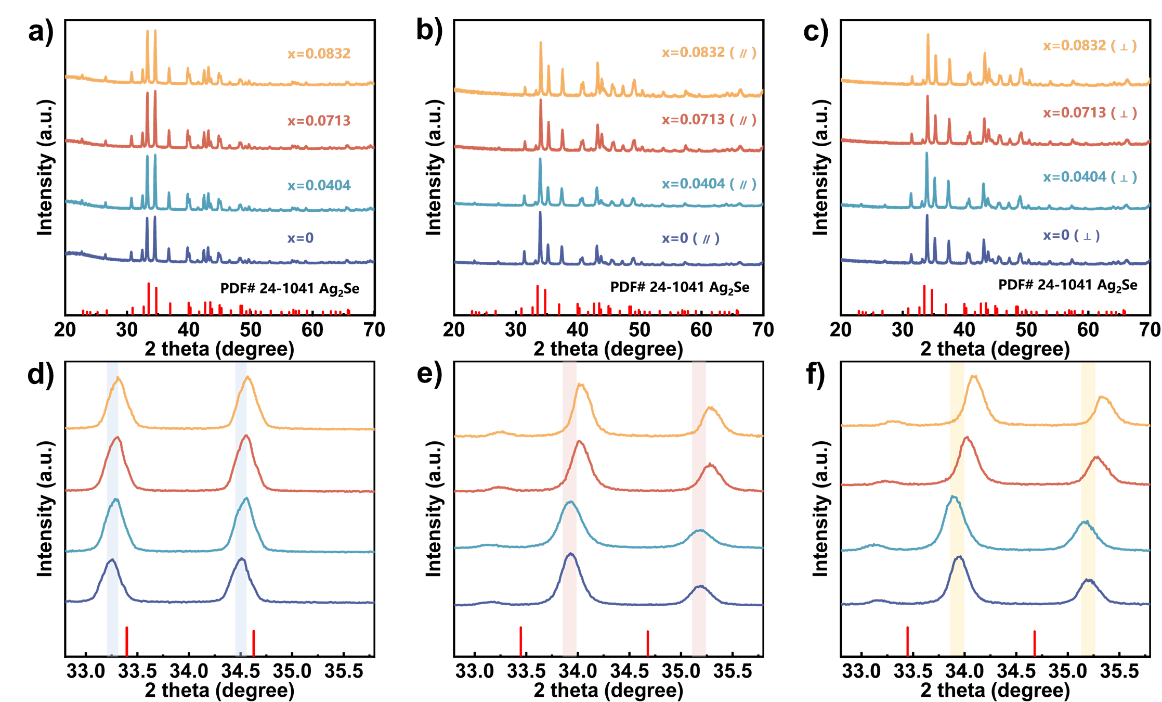
*

**Figure S8.** XRD patterns of Ag_2-x_In_x_Se samples. (a) As-synthesized powder; (b) SPS-sintered bulk measured parallel to the pressing direction; (c) SPS-sintered bulk measured perpendicular to the pressing direction. (d-f) Magnified views of the corresponding regions in (a-c).

*
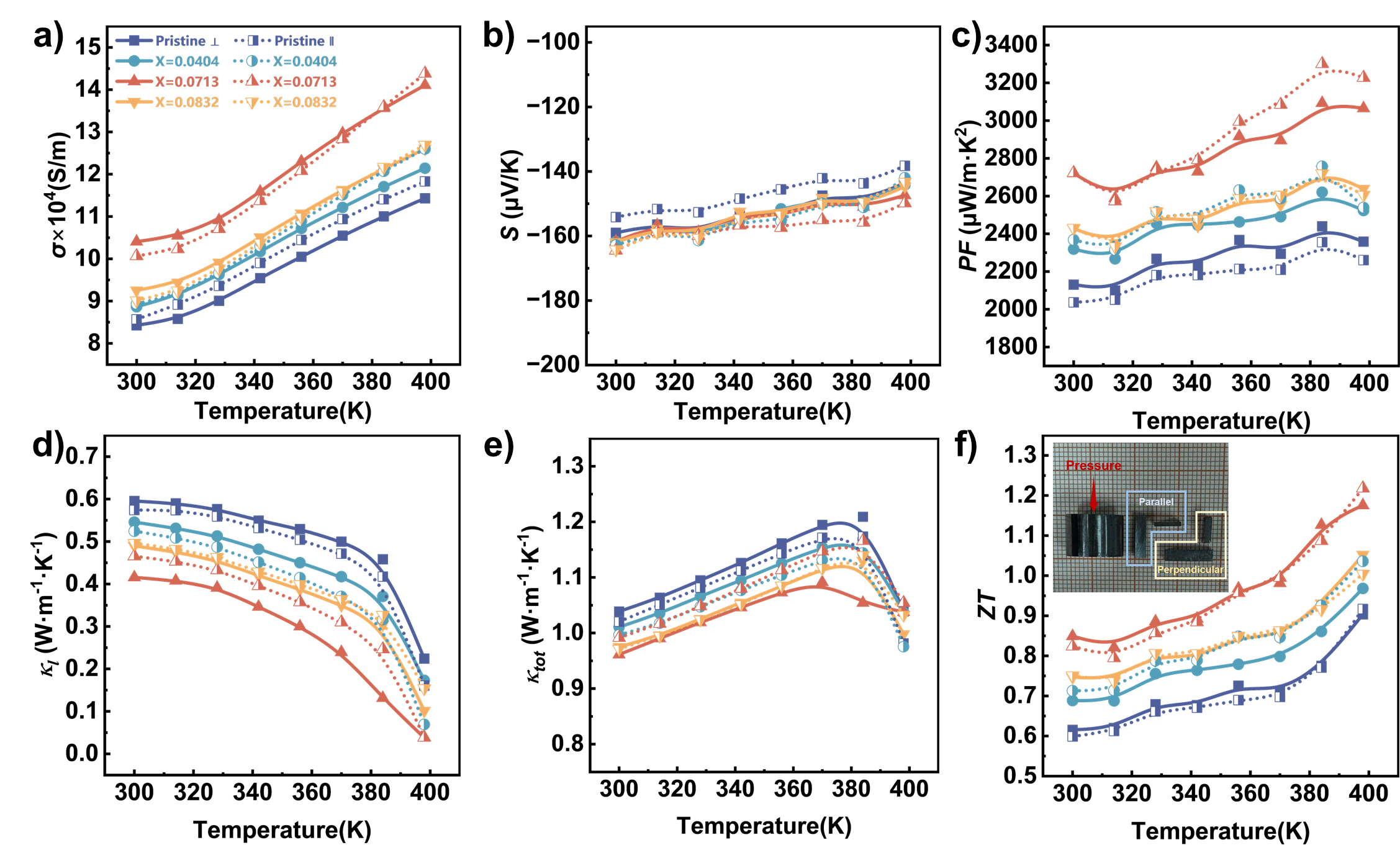
*

**Figure S9**. Thermoelectric measurements for both perpendicular and parallel direction to SPS. Temperature dependence of (a) *𝜎* ; (b) *S* ; (c )*PF* ; (d) *κ_l_* ; (e) *κ_tot_* ; (f) *ZT* ,with a photograph for specimens used for measurement.


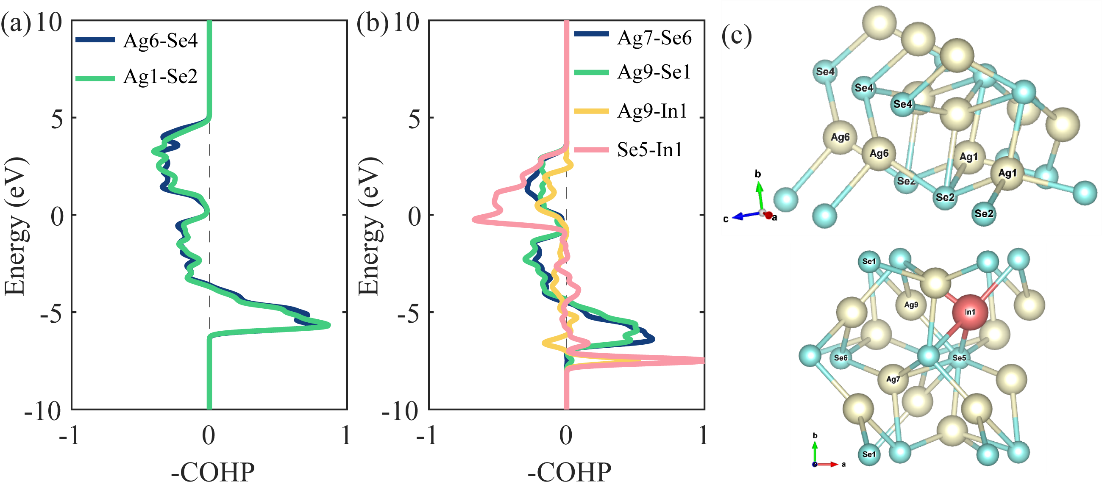


**Figure S10.** The corresponding crystal structures of Ag₂Se and Ag₁.₉₂₈₇In₀.₀₇₁₃Se.


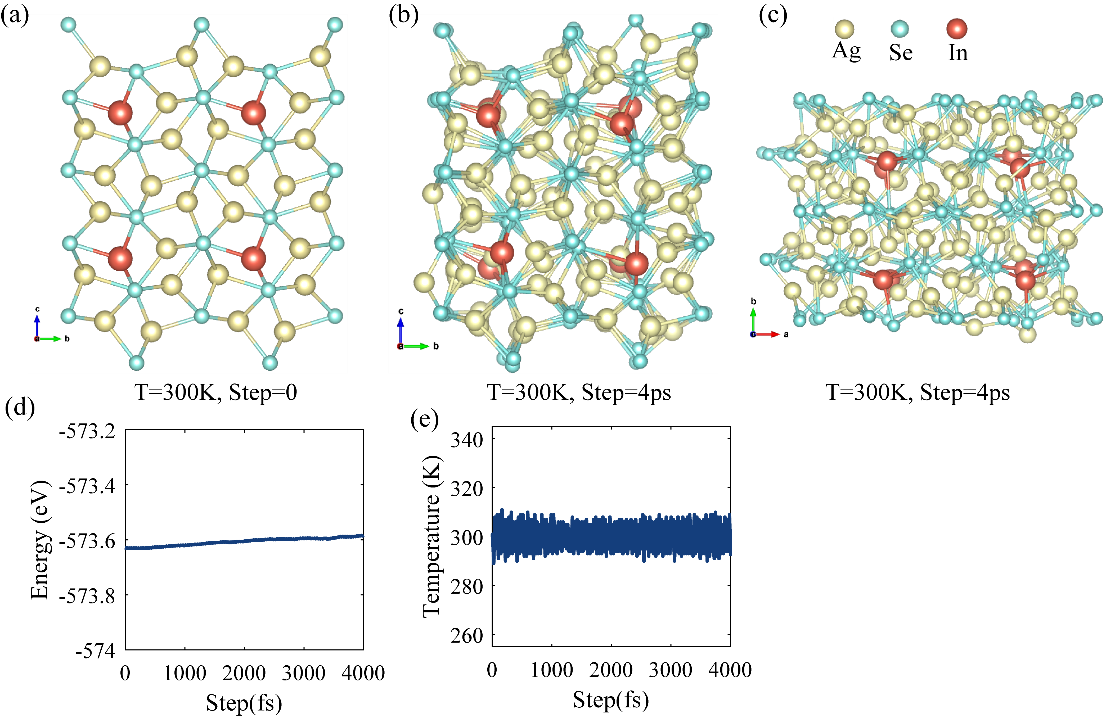


**Figure S11.** Atomic snapshot of Ag_1.9287_In_0.0713_Se obtained via ab initio molecular dynamics (AIMD) simulations at 300 K, showing (a) the intrinsic structure in top view, (b)-(c) the structure in side views, and (d) the free energy fluctuation at different time points over a total simulation time of 4 ps.


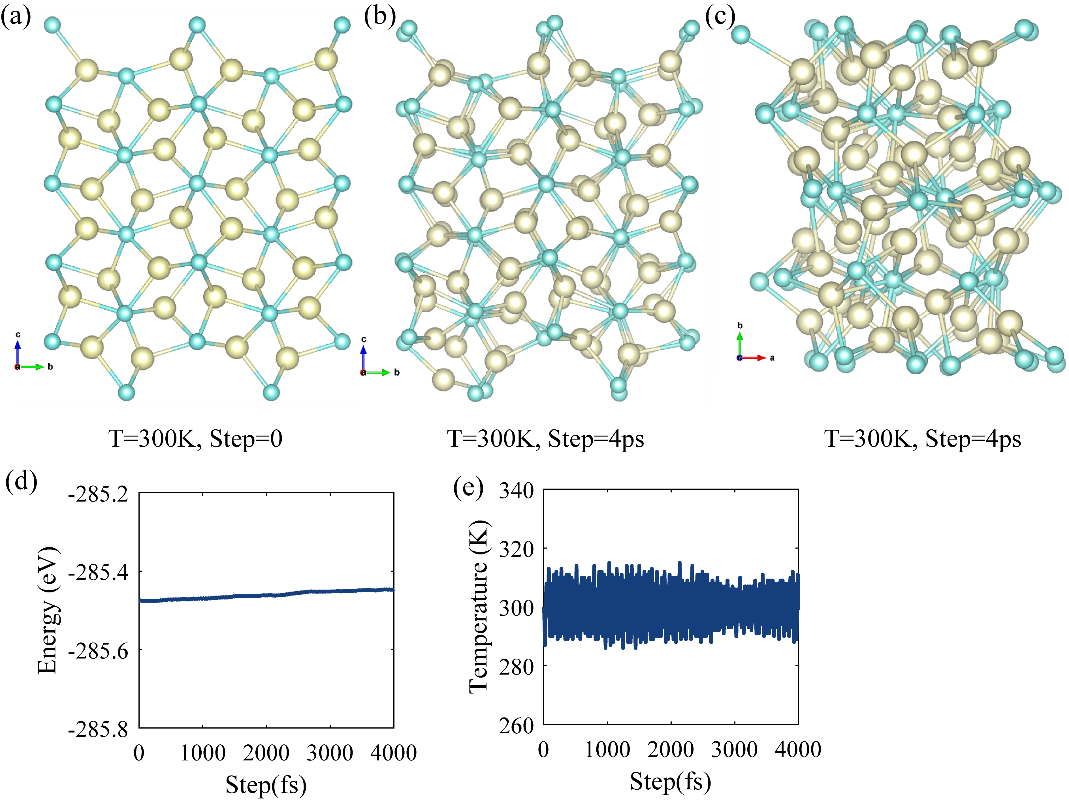


**Figure S12.** Snapshot of Ag₂Se from AIMD calculations at 300 K: (a) Top view and (b)-(c) Side views of the intrinsic structure; (d) Time-dependent fluctuation of free energy over a total simulation duration of 4 ps.

**
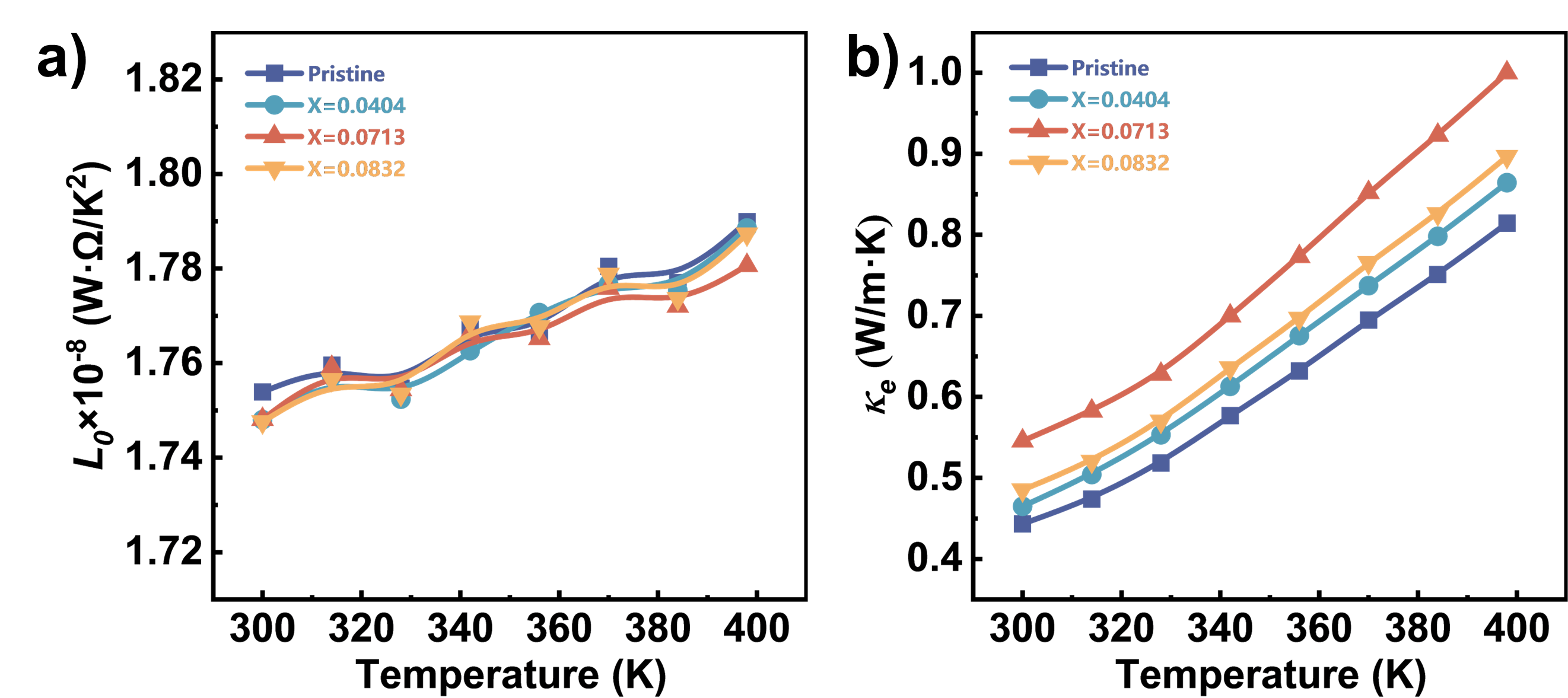
**

**Figure S13.** Temperature dependence of a) Lorentz number (*L_0_*) and b) carrier thermal conductivity (*κ_e_*) of Ag_2-x_In_x_Se pellets.


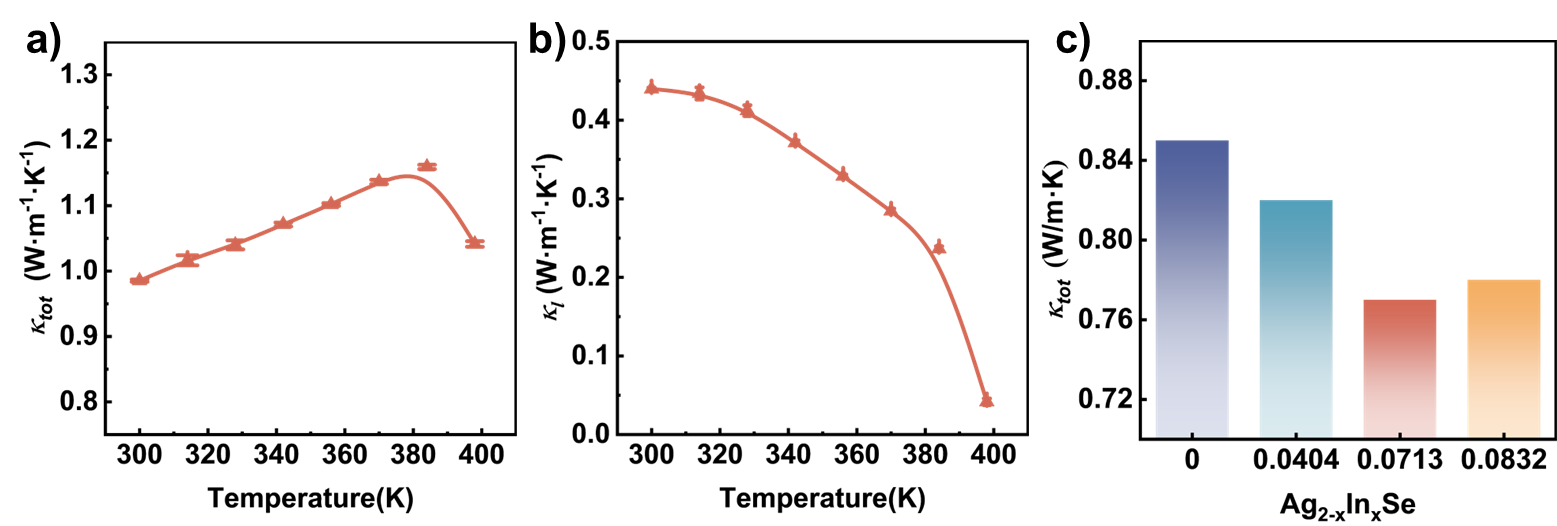
**Figure S14**. a) *κ_tot_* and b) *κₗ* of the Ag_1.9287_In_0.0713_Se pellets via NETZSCH Laser Flash Analysis (LFA) c) Room-temperature thermal conductivity measurements of Ag_2-x_In_x_Se pellets via TTR.


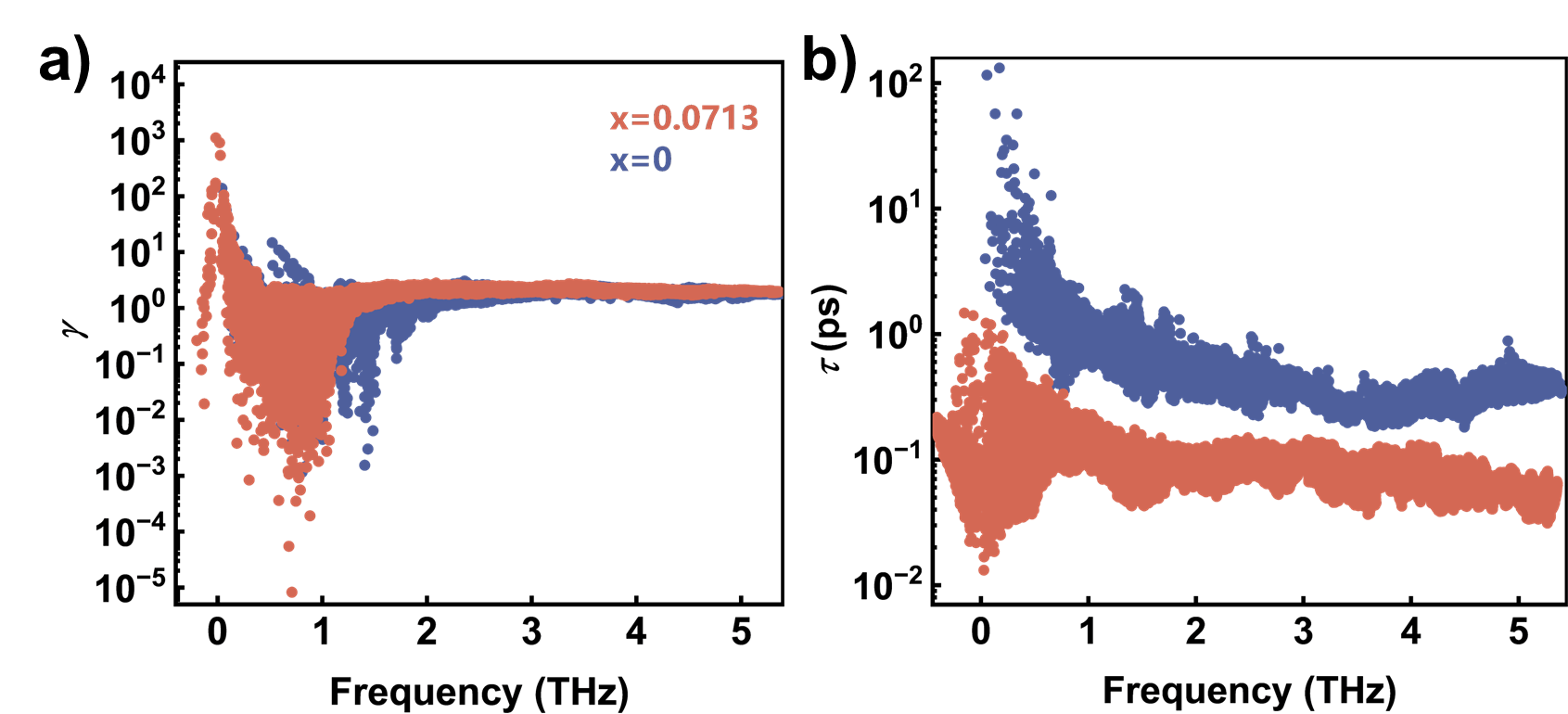


**Figure S15**. (a) Grüneisen parameters and (b) phonon relaxation times of Ag_2-x_In_x_Se.


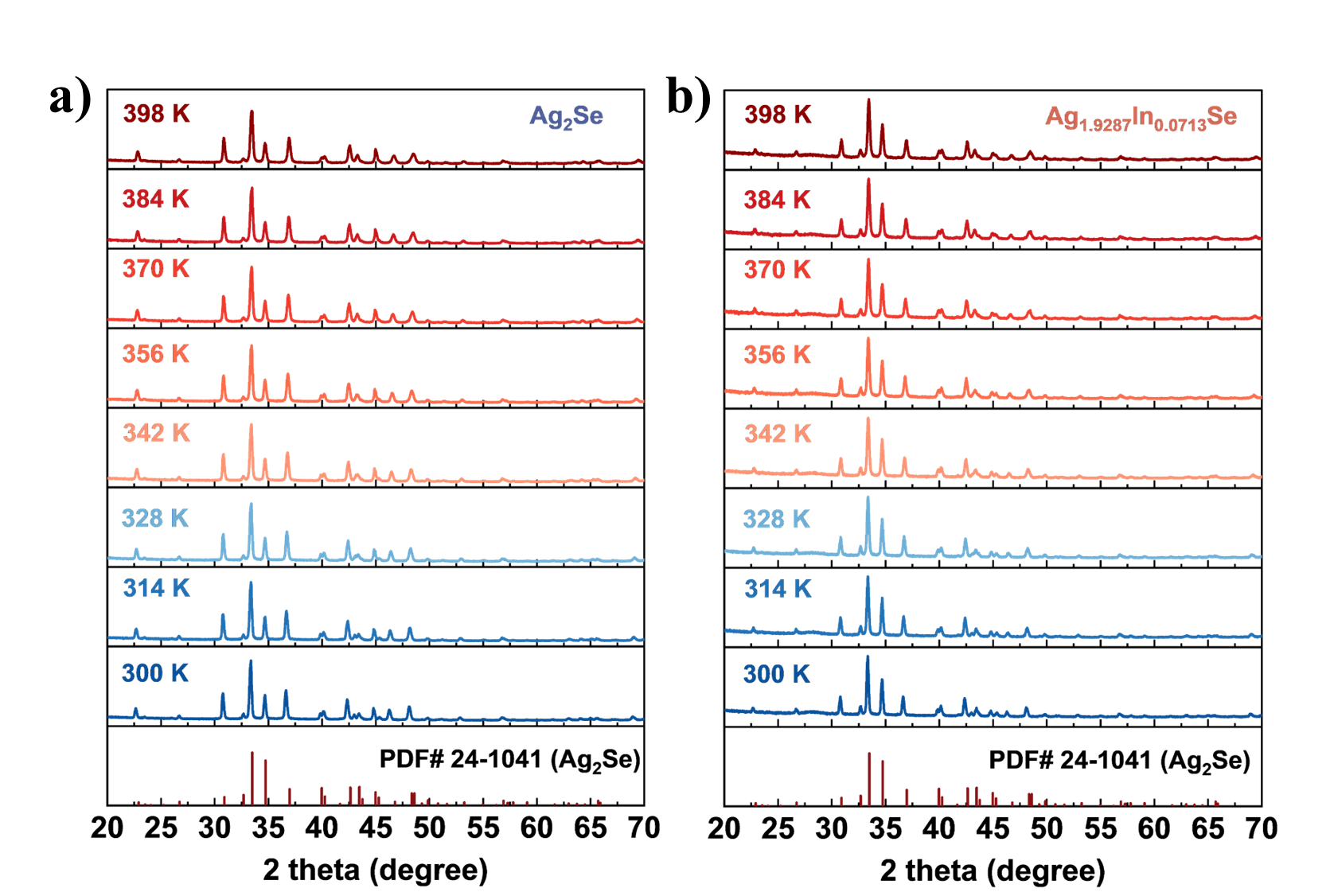


**Figure S16.** Temperature dependent XRD patterns of a) Ag_2_Se, and b) Ag_1.9287_In_0.0713_Se pellets.


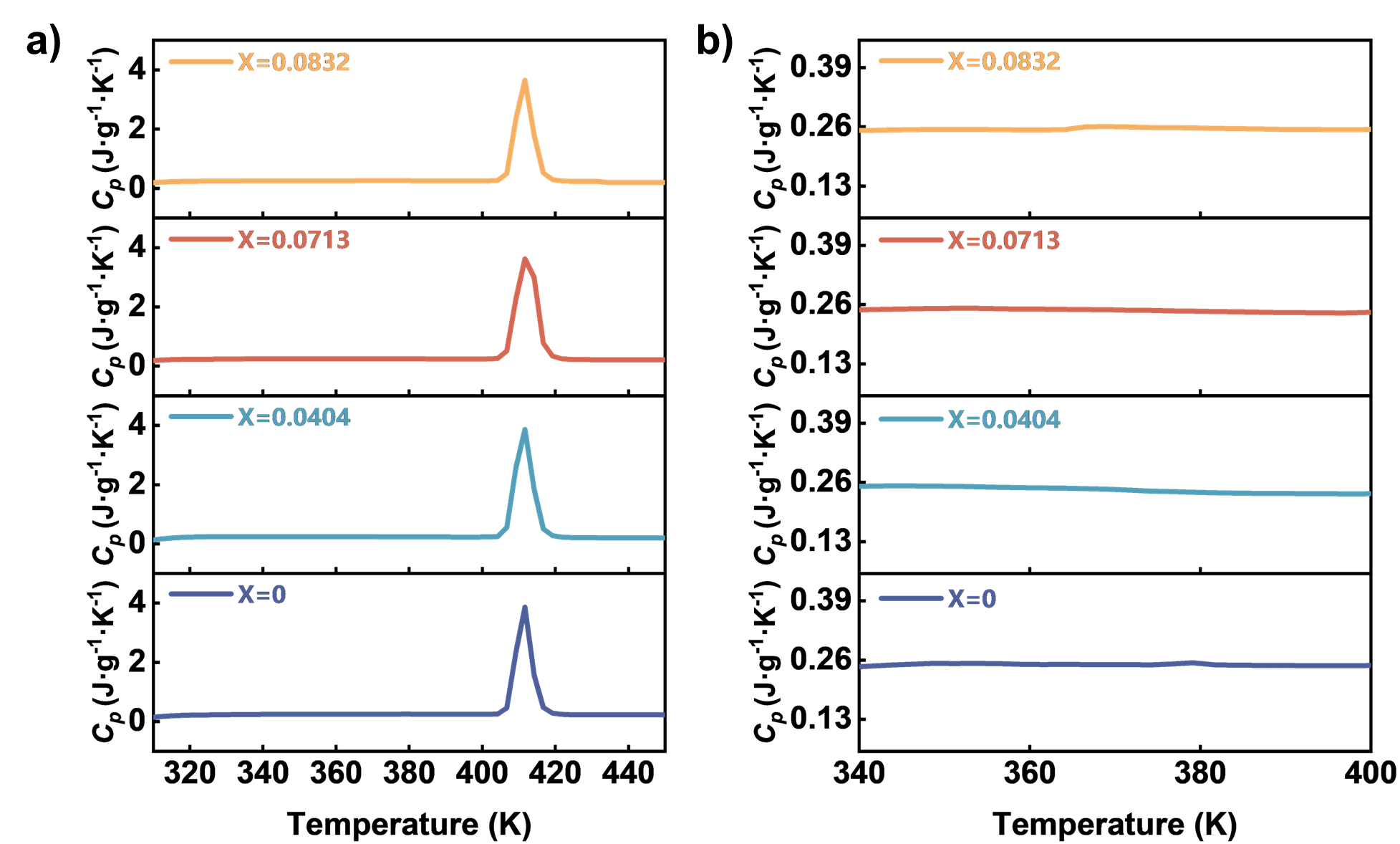


**Figure S17.** DSC curves of Ag_2-x_In_x_Se powder.

*
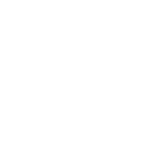

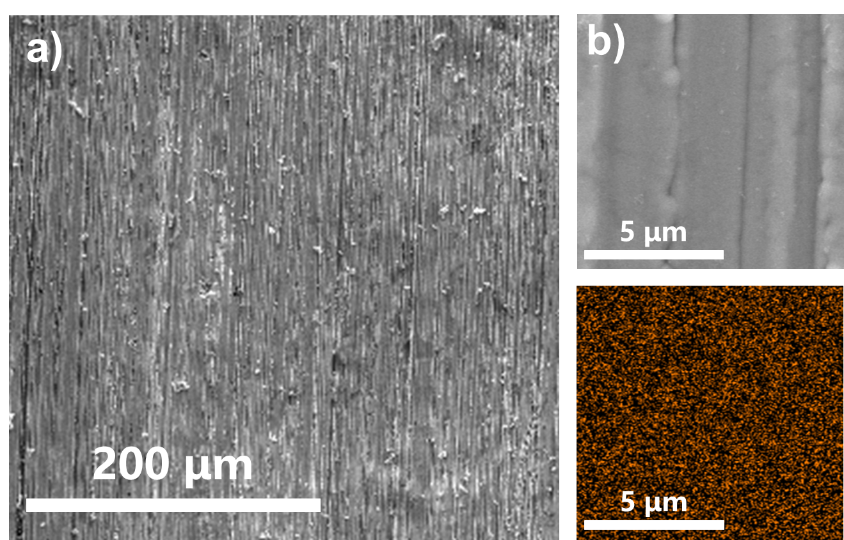
*

**Figure S18.** a) SEM images of Ag_1.9287_In_0.0713_Se n-type thermoelectric legs and b) Local magnification and EDS mapping.

*
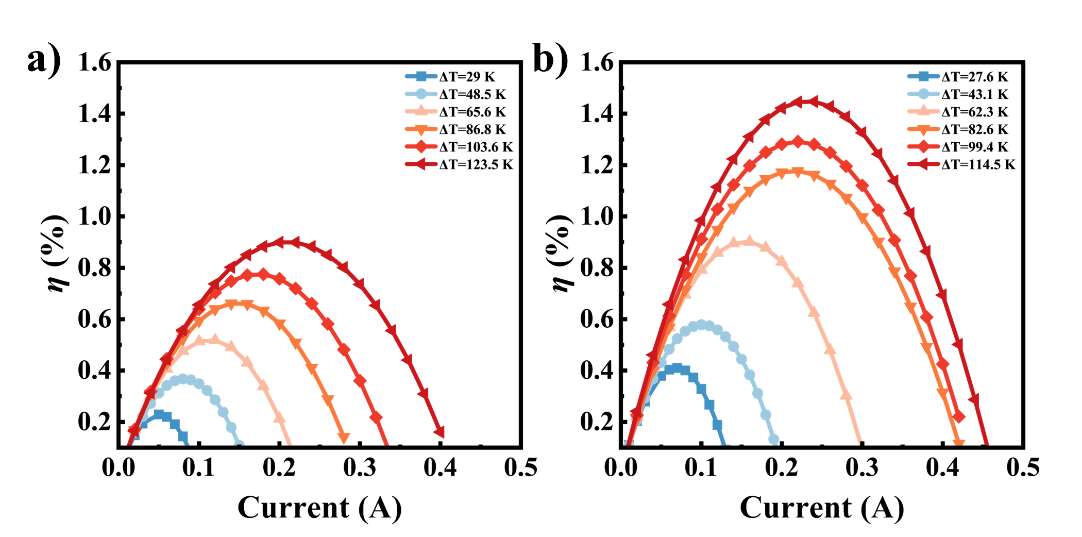
*

**Figure S19.** Measured energy conversion efficiency of of multi-leg devices using a) pristine Ag_2_Se and b) Ag_1.9287_In_0.0713_Se as n-type thermoelectric legs.

**
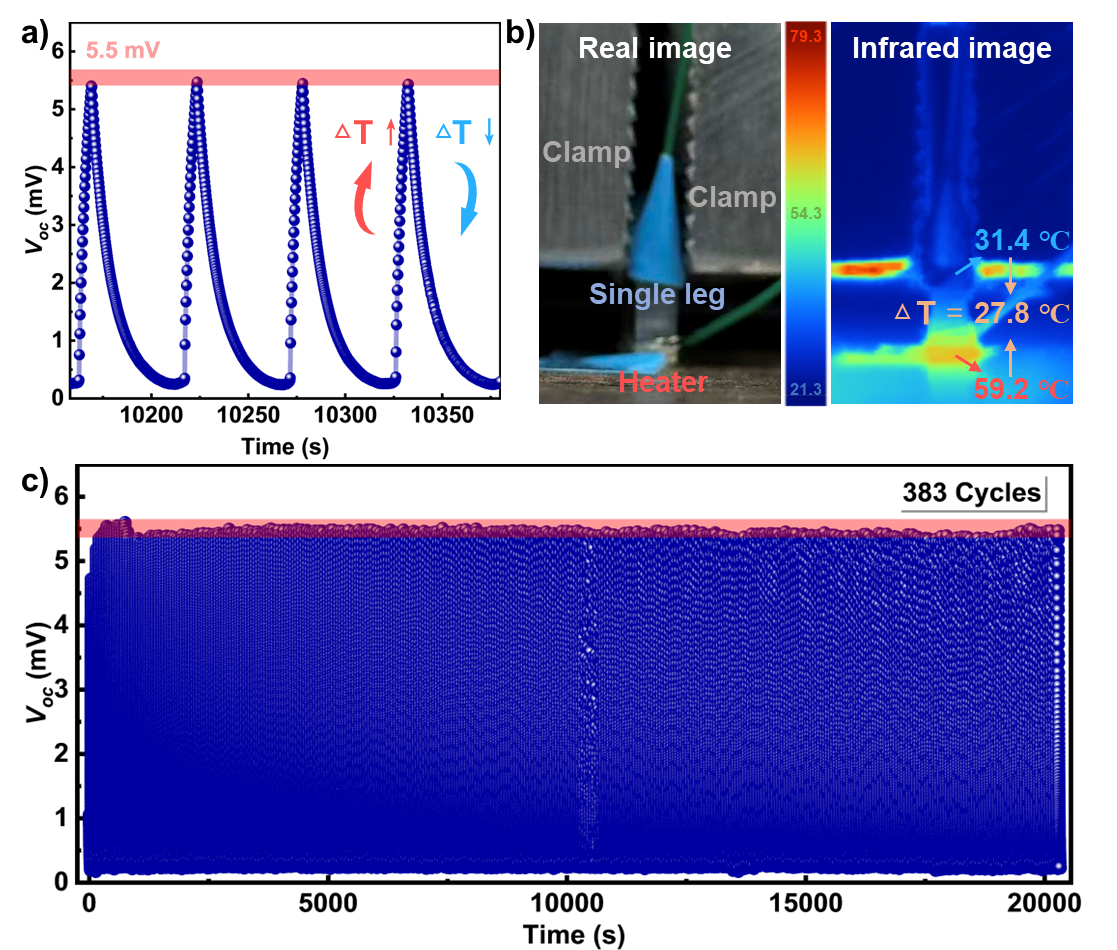
**

**Figure S20**. Open-circuit voltage of Ag_1.9287_In_0.0713_Se single-leg device under 383 times thermal cycles: (a) Random four times thermal cycles; (b) Test diagram of single-leg thermal cycle and infrared thermal imaging; (c) Single-leg thermal cycle 383 times.


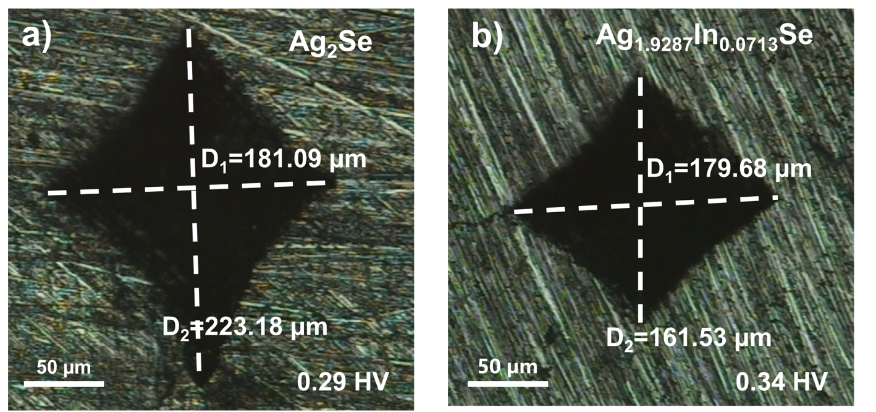


**Figure S21**. Nanoindentation of Ag_2_Se and Ag_1.9287_In_0.0713_Se.

**Table S1.** ICP-OES results of Ag and In contents in the doped-sample in a synthesis.

| Sample | Ag/mol | In/mol |
| --- | --- | --- |
| Ag_1.9596_In_0.0404_Se | 0.01365 | 0.000635 |
| Ag_1.9287_In_0.0713_Se | 0.013323 | 0.001019 |
| Ag_1.9168_In_0.0832_Se | 0.012364 | 0.001585 |

|  | Ag_2_Se | Ag_1.9596_In_0.0404_Se | Ag_1.9287_In_0.0713_Se | Ag_1.9168_In_0.0832_Se |
| --- | --- | --- | --- | --- |
| a | 4.33465±0.000196 | 4.33298±0.000095 | 4.33281±0.000144 | 4.33233±0.000088 |
| b | 7.06545±0.000311 | 7.06337±0.000145 | 7.06173±0.000229 | 7.06063±0.000152 |
| c | 7.76761±0.000345 | 7.76492±0.000163 | 7.76217±0.000254 | 7.76100±0.000185 |
| χ^2^ | 1.09 | 1.25 | 1.04 | 0.96 |

**Table S2**. Standard deviation for the unit cell parameters and χ^2^.

**Table S3.** Relative density of Ag_2-x_In_x_Se pellets obtained from absolute values measured by the Archimedes’ method. The measured pellet density was compared with the density of Ag_2_Se, 8.24 g/cm^3^.

| Sample | Measured density (g/cm^3^) | Relative density |
| --- | --- | --- |
| Ag_2_Se | 8.217 | 99.72% |
| Ag_1.9596_In_0.0404_Se | 8.195 | 99.45% |
| Ag_1.9287_In_0.0713_Se | 8.202 | 99.54% |
| Ag_1.9168_In_0.0832_Se | 8.238 | 99.98% |

**Table S4.** Room temperature longitudinal (*v_l_*) and shear (*v_t_*) acoustic sound velocities of Ag_2-x_In_x_Se pellets.

| Acoustic sound velocity (m/s) | *v_l_* | *v_t_* | *v_avg_* |
| --- | --- | --- | --- |
| Ag_2_Se | 1671.43 | 801.37 | 901.08 |
| Ag_1.9596_In_0.0404_Se | 1035.09 | 766.23 | 824.75 |
| Ag_1.9287_In_0.0713_Se | 786.67 | 797.30 | 793.69 |
| Ag_1.9168_In_0.0832_Se | 850.00 | 793.33 | 810.53 |

**
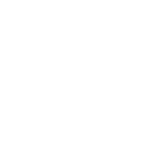
Table S5**. key parameters of multi-leg devices.

| Thermoelectric Device | | Leg Length | Cross-sectional Area | Contact Materials | Contact Resistance |
| --- | --- | --- | --- | --- | --- |
| Ag_1.9287_In_0.0713_Se leg | Commercial  p-type Bi₀.₅Sb₁.₅Te₃ leg | 2.5 mm | 1.96 mm^2^ | Cu | 0.043 Ω |
| Ag_2_Se leg | Commercial  p-type Bi₀.₅Sb₁.₅Te₃ leg |  |  |  | 0.042 Ω |

**References:**

[1] Y. Zhang, Z. Li, S. Singh, A. Nozariasbmarz, W. Li, A. Genç, Y. Xia, L. Zheng, S. H. Lee, S. K. Karan, G. K. Goyal, N. Liu, S. M. Mohan, Z. Mao, A. Cabot, C. Wolverton, B. Poudel, S. Priya, *Adv. Mater.* 2023, **35**, 2208994.

[2] Y. Liu, M. Calcabrini, Y. Yu, A. Genç, C. Chang, T. Costanzo, T. Kleinhanns, S. Lee, J. Llorca, O. Cojocaru-Mirédin, M. Ibáñez, *Adv. Mater.* 2021, **33**, 2106858.

[3] Y. Xiao, C. Chang, Y. Pei, D. Wu, K. Peng, X. Zhou, S. Gong, J. He, Y. Zhang, Z. Zeng, L.-D. Zhao, *Physical Review B* 2016, **94**, 125203.

[4] G. Kresse, J. Hafner, *Physical Review B* 1994, **49**, 14251.

[5] A. Togo, I. Tanaka, *Scr. Mater.* 2015, **108**, 1.

[6] V. L. Deringer, A. L. Tchougréeff, R. Dronskowski, *The Journal of Physical Chemistry A* 2011, **115**, 5461.

[7] B. Li, M. Li, H. Qi, X. Zu, L. Qiao, H. Xiao, *Crystals* 2023, **13**, 1586.

[8] Y. Suzuki, H. Nakamura, *Physical chemistry chemical physics : PCCP* 2015, **17**, 29647.
